# Facial and body sexual dimorphism are not interconnected in the Maasai

**DOI:** 10.1186/s40101-021-00276-8

**Published:** 2022-01-07

**Authors:** Marina L. Butovskaya, Victoria V. Rostovtseva, Anna A. Mezentseva

**Affiliations:** 1 Institute of Ethnology and Anthropology, Russian Academy of Sciences, Moscow, 119334 Russia; 2 The National Research University Higher School of Economics, Moscow, 101000 Russia; 3 Russian State University for the Humanities, Moscow, 125047 Russia

**Keywords:** Sexual dimorphism, Facial geometric morphometrics, fWHR, Body height, Facial dimorphism, Body dimorphism, Sex differences, Maasai

## Abstract

**Background:**

In this paper, we investigate facial sexual dimorphism and its’ association with body dimorphism in Maasai, the traditional seminomadic population of Tanzania. We discuss findings on other human populations and possible factors affecting the developmental processes in Maasai.

**Methods:**

Full-face anthropological photographs were obtained from 305 Maasai (185 men, 120 women) aged 17–90 years. Facial shape was assessed combining geometric morphometrics and classical facial indices. Body parameters were measured directly using precise anthropological instruments.

**Results:**

Sexual dimorphism in Maasai faces was low, sex explained 1.8% of the total shape variance. However, male faces were relatively narrower and vertically prolonged, with slightly wider noses, narrower-set and lower eyebrows, wider mouths, and higher forehead hairline. The most sexually dimorphic regions of the face were the lower jaw and the nose. Facial width-to-height ratio (fWHR), measured in six known variants, revealed no significant sexual dimorphism. The allometric effects on facial traits were mostly related to the face growth, rather than the growth of the whole body (body height). Significant body dimorphism was demonstrated, men being significantly higher, with larger wrist diameter and hand grip strength, and women having higher BMI, hips circumferences, upper arm circumferences, triceps skinfolds. Facial and body sexual dimorphisms were not associated.

**Conclusions:**

Facial sex differences in Maasai are very low, while on the contrary, the body sexual dimorphism is high. There were practically no associations between facial and body measures. These findings are interpreted in the light of trade-offs between environmental, cultural, and sexual selection pressures.

**Supplementary Information:**

The online version contains supplementary material available at 10.1186/s40101-021-00276-8.

## Background

The idea that human facial and body sexual dimorphism are both products of sexual selection has a long history (at least 150 years), dating back to Darwin’s seminal book “The Descent of Man, and Selection in Relation to Sex” [1]. Since then, evolutionary biologists have been trying to detect which factors determine the strength of mate choice and intensity of sexual selection in each sex, and the progress made in this direction is really impressive [2–7]. Sexual dimorphism has been interpreted as a product of the exposure to sex hormones (testosterone, estrogens) [8–17], and currently some studies pointed to sex differences in genes expression in human tissues [18] and sex differences in immune responses to pathogens, including COVID-19 [19]. Masculinity in males and femininity in females have been viewed as true signals of immune qualities, providing better prospects for survival and reproduction [20–23], and at least some aspects of immune function during early adolescence may positively predict sexually dimorphic 3D face shape in both men and women [24]. The role of ecological [25, 26] and cultural factors, particularly, norms, traditions, economy [27, 28] in the emergency of population variations in facial and body sexual dimorphism in humans has been also an object of intensive discussions [3, 8, 29]. Cranial (facial) form and robusticity demonstrate substantial variation related to climate and ecology (geography) [30], and sexual dimorphism varies between races and ethnics [31–38].

Male facial, as well as body masculinity has been frequently viewed as cues to good health and “good genes” (i.e., genes promoting health) [39], as producing and metabolizing testosterone is costly (and might lead to higher oxidative stress) [40]. Some studies, indeed, demonstrate that high testosterone levels increase muscularity and body weight [41], as well as positively associated with facial masculinity [42, 43]. Studies conducted on Caucasian samples revealed certain relationships between facial and body traits. Face growth occurs in concordance with body growth [44], and according to allometry effects, facial shape is expected to be associated with body size [45, 46]. In modern western populations, for example, in Germans, taller men were reported to have longer, narrower jaws and wider/fuller lips, besides, changes in male facial shape were more strongly associated with physical strength, which in turn was related to perceived masculinity [45]. However, today, it is known that relation between facial shape and body size is far more complicated and subjected to large population variation [38].

Masculine men report less interest in child rearing and higher rates of short-term relationships [47, 48]. It is hypothesized that women select masculine male partners when the costs of reduced paternal investment are compensated by some genetic benefits to future offspring [49]. Some studies suggest that short-term relationships may be beneficial in populations with high pathogen pressure [50, 51]. Mating or reproductive success may be associated with development of such sexually dimorphic traits as muscularity, height, and facial and vocal masculinity [52]. Physical strength is an influential trait in male-male contest competition [53]. Some authors provided evidence for a stronger influence of male-male competition in modern western society. Based on data from young age cohort (18 to 34 years), it was demonstrated that more physically dominant men reported about higher mating success compared to more sexually attractive men [54]. Body size and androgen-dependent traits are of great importance for intra-sexual competition as well [55]. It may be asked in this context whether masculinity in males has being even more strongly positively selected in polygynous populations with high natural fertility profile, such as Maasai, Datoga, Ariaal, Turkana, or other East African pastoralists, since intrasexual competition between males in these populations has been really high. In relation to our study, it makes sense to note that Maasai, as Nilotic people of East Africa, are rather tall and skinny. The level of testosterone in men from such populations is usually lower, compared to men of Caucasian origin from western populations [56]. On the other hand, testosterone level does not differ much in different age groups, in contrast to men from western populations [57, 58], and their reproduction life extends to 70^th^ year and even further.

Physical height has a well-documented impact on human social status in western societies. Taller men in western societies have been perceived as stronger, smarter, and more dominant [59]. They are also known to have higher reproductive success [60], although the curvilinear effect on reproductive success in men should be taken into consideration [61]. Height may influence mate choice, particularly, “man taller than women” norm is widely followed both by men and women [62]. However, it is important to mention that this norm is far from being universal [63–65].

One of the facial parameters, which has attracted a special attention of anthropologists and behavioral scientists today, is the facial width-to-height ratio (fWHR), defined as the ratio of the bizygomatic width to the so-called upper facial height (measured from Nasion to Prosthion, or their soft-tissue approximations) [33, 66]. This index associates with facial masculinity and has been viewed as sexually dimorphic, being higher in males than females [33, 67–72]. Weston and colleagues [73] speculated that sexual dimorphism in the fWHR evolved via female choice as an attractive trait. However, a number of studies do not find any differences in fWHR between men and women [74–79], or report even higher fWHR in women compared to men [37].

It has been hypothesized that the fWHR is part of an evolved cueing system of intra-sexual threat and dominance in men [80]. Numerous studies connect the fWHR with dominance, threat, fighting [66, 81–86] (however, see: [87]), reproductive success [88, 89], and deception [90]. It was also hypothesized that fWHR was subjected to positive sexual selection in the whole genus Homo [91], and generally in higher primates [73]. fWHR may serve as honest cue to males’ ability to cooperate in intergroup conflicts, hence selected as such indicator in intrasexual selection [92]. Particularly, it was demonstrated by Stirrat and Perrett [93] that fWHR in men associates with increase in cooperation with other in-group members during intergroup competition. Men with wider faces were more self-sacrificing and helpful to their group, while competing rivalry group. These findings make sense in the light of parochial altruism hypothesis [94–96].

However, the association between fWHR and behavioral traits may be much more complex, ecological, social, developmental factors should be considered, and the evolutionary hypothesis related to fWHR selection in humans and apes must be taken with caution. Wilson with co-authors reported the absence of sexual dimorphism on fWHR in chimpanzees, as well as no association between fWHR and dominance in this species [97], several studies failed to establish association between fWHR with aggression and dominance in men [98–101]. No associations between testosterone and fWHR [102–105], and between the androgen receptor gene polymorphism and fWHR [103] in men were demonstrated.

In accordance with “good genes” hypothesis, significant positive association between major histocompatibility complex (MHC) heterozygosity (an indirect measure of improved immunity) and facial attractiveness has been reported in young British and Australian men [106, 107], but not women [107, 108]. Significant positive correlations between immune response (cytokine response before and after immune stimulation), attractiveness, and health were also found in African men [109]. However, some studies have failed to find a relationship between attractiveness and health [110, 111]. Contradictive results may be due to variations in immunity parameters. The data on various parameters of immunity in relation to differences in androgen levels in men from well-nourished Western society, presented by Nowak with co-authors [112], suggest that androgens may act as immunomodulators rather than immunosuppressants. The immune-androgen interaction may be highly affected by a number of physiological or ecological factors [112], including the availability of nutritional resources, the intensity pathogen exposure, the extrinsic mortality risk [113], various stresses [108], and living conditions [114].

Sexual dimorphism can be studied on the basis of various parameters: musculature, facial shape, body height, etc. Below, these variables and their relationships will be presented.

In this paper, we address the questions of universality of facial sexual dimorphism in modern humans (Homo sapiens), and its’ association with body sexual dimorphism. For these purposes we investigate sex differences in full facial shape and linear facial measures in association with body sexual dimorphism in Maasai, the traditional seminomadic East African Nilotic population of Northern Tanzania. The Maasai population of Ngorongoro Conservation Area was selected because they remained highly traditional in their economy (pastoralists), ecology (semi-nomadic, with traditional way of living and exposed to regular threat from wild animals, including regular attacks of lions, hyenas, and other carnivorous on cattle and people), and culture (preserving the age-set system, clan organization, practicing polygyny, and high fertility).

## Methods

### Study population

The Maasai are the Maa-speaking pastoral people of Tanzania. According to a census conducted in 2007, their population in the Ngorongoro Conservation Area (NCA) was 70,000 [115]. The Maasai culture and social structures are highly conservative [116]. Maasai society is structured around two major social institutions: the age-set system and the clans [117]. Today, the Maasai of the NCA are exclusively pastoralists, as far as any sort of agricultural activity is not allowed inside the habitat [118]. Currently, about half of the married men are in polygynous relationships with an average of 2.8 wives per man [119]. A system of territorial groups (sections), clans, and age-sets provide the basis of social and economic cooperation.

The age-set system remained to be important in men’s life. Adjacent age-sets are in lifelong political and ritual opposition and competition. Every 15 years, a new age-set is opened. All boys of suitable age become circumcised during this period and join a group of “ilmurran” (“junior warriors”). Murrans spend time traveling, herding, and feasting in the bush. They also act as warriors, defend local households from raids of neighboring tribes (Datoga and Sukuma), and in turn steal cattle from them [118]. Those who are more successful in these activities gain higher social prestige, and this may facilitate access to female mating partners. Around the 30–35 years of age men enter the stage of junior elders, and according to traditional norms men are allowed to marry [117]. Currently, however, men started to marry earlier; however, they still follow a number of restrictions, particularly do not take food with their wives until they officially join the junior elders stage. Since this time physical strength and competition do no longer of primary importance, and men gradually gaining social respect and power. Fifteen years later, they become senior elders, and gain significant authority over decisions regarding resource use, livestock, water resources, mutual aid, and other issues [120].

### Study sample

Our data were collected in 2016 in and around Endulen village, located in NCA. All Maasai who participated in this study were living in traditional Maasai households, practicing pastoralism and traditional cultural norms. The total sample size was 305 individuals (185 men and 120 women) with an age range between 17 and 90 years [121].

The study was conducted according to the principles of the Declaration of Helsinki. The population studied was illiterate and therefore a written consent could not be obtained. The participants gave verbal consents and were told that their participation was voluntary and that they could withdraw from the study at any time. The study protocol and consent procedure received ethical approval from the Ethics Committee of Moscow State University; research permit was obtained from the Tanzania Commission for Science and Technology (COSTECH) and administration of Ngorongoro Conservation Area (NCA).

The data that support the findings of this study are available from the corresponding author upon reasonable request.

### Facial photographs

Each participant was photographed in full-face perspective. Subjects were seated at 1.80 m distance to the camera and were instructed to look straight into the lens (Nikon D90, 70 mm lens equivalent to 105 mm for 35 mm film) while maintaining a neutral facial expression. A scale bar (in cm) was included in each image. Faces were positioned visually according to the Frankfort Horizontal Plane (FH) with the lens at eye height.

### Geometric morphometrics

Facial shape analysis was held using geometric morphometrics [122]. Seventy-one facial landmarks and semi-landmarks were manually placed on each photograph using tpsDig2 2.17 [123]. Landmarks’ positions were set based on the configuration developed by Windhager et al. [45], which has been already used in our earlier studies [37, 121, 124]. This configuration included 37 landmarks, which are known to be classical anthropometrical approximations to cranio-facial and soft-tissue facial shape determinants [33, 45, 66, 125, 126] as well as 34 semilandmarks, used for covering facial outline, eye-brows and lips shapes.

To ensure reliability of the landmarks’ digitalization, two independent observers were invited to place all 71 landmarks and semilandmarks on 40 randomly selected photographs (20 male and 20 female faces). The repeatability of landmarks’ digitalization was assessed by means of geometric morphometrics as the ratio of among-individual variance component to the sum of among-individual and measurement error components [127], using “vegan” package for R (adonis() function with Euclidian method) [128]. The inter-observer agreement was 0.91. We considered the method reliable enough to proceed with manual digitalization of landmarks by one of the observers.

Facial centroid size as one of the major measures of allometry [46, 127] was calculated using «shapes» package for R [129] after scaling all facial configurations to centimeters.

All facial configurations were standardized for the position, orientation, and scale by Generalized Procrustes superimposition together with sliding semilandmarks using minimum bending energy criterion in “geomorph” package for R [130].

Since facial asymmetry estimation was not among the goals of the present study, all facial configurations were symmetrized [131] in order to reduce possible distortion due to head positioning in the 2D projection. Symmetrization was performed in R [132], using basic functions, and those developed by Claude [133].

Association of the facial shape with sex, age, and body mass index (BMI) were tested by multivariate analysis of variance using “vegan” package for R (adonis() function with Euclidian method) [128].

Visualization of the results of morphometric analysis was realized using different methods. Thin-plate deformation grids with pairwise comparison of target configurations were made in R using functions developed by Claude [133], and adjusted by authors according to the purposes of the present study. Geometric morphometric morphs were visualized by unwarping and averaging photographs in tpsSuper 2.04 [45, 123].

### Classical facial metrics

**Fig. 1:**
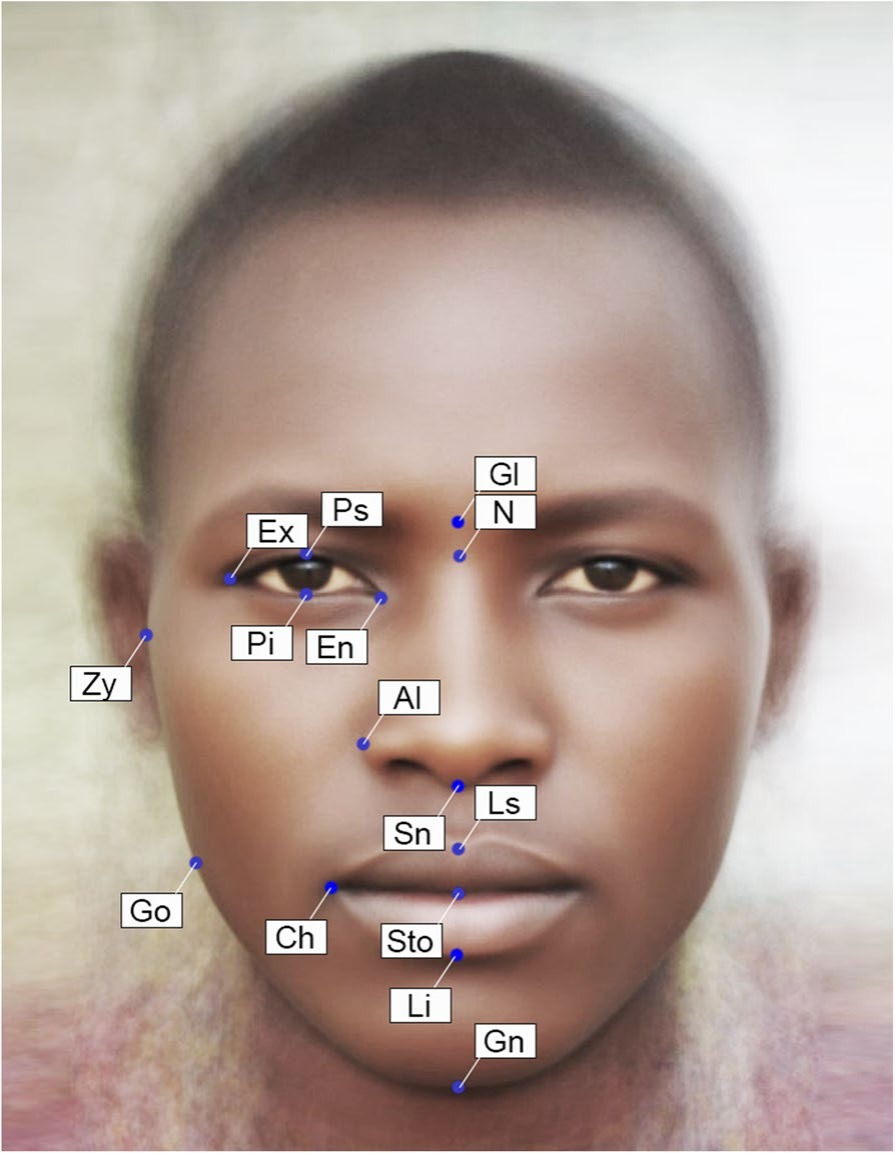
Facial landmarks used in the classical facial metrics. Averaged Maasai portrait. Single landmarks: Gl—*glabella* (midpoint between the center points of the lower eyebrows' hairlines); N—*nasion* (midpoint between the highest points of the eyelids); Sn—*subnasale* (the midpoint of the base of the columella); Ls—*labiale superius* (the outermost point on the upper lip vermillion in the midsagittal plane); Sto—*stomion* (the point in the midsagittal plane where upper and lower lips vermillions meet); Li—*labiale inferius* (the outermost point on the lower lip vermillion in the midsagittal plane); Gn—*gnathion* (the lowest point of the chin in the midsagittal plane). Paired landmarks: Zy—*zygion* (the most lateral point of the zygomatic arch); Go—*gonion* (the most inferior and lateral point on the external angle of the mandible); Ch—*cheilion* (the outer corner of the mouth); Al—*alare* (the most lateral point of the nasal wing); Ex—*exocanthion* (the lateral point of the eye fissure); En—*endocanthion* (the medial of the eye fissure); Ps—*palpebrale superius* (the highest point of the free margin of the upper eyelid); Pi - *palpebrale inferius* (the lowest point of the free margin of the lower eyelid)

Classical facial indices, which are known to demonstrate certain degree of sexual dimorphism in humans, were calculated based on coordinates of facial landmarks after Procrustes superimposition. These indices included upper width-to-height ratio (upper fWHR); total width-to-height ratio (total fWHR); lower width-to-height ratio (lower fWHR); cheekbone prominence; and mandibular and nasal indices, mouth shape, mouth-face index, and height-to-width ratio of the eye [37, 66, 72]. The locations of the landmarks used for calculation of these indices are presented in Fig. 1. Details on the estimated facial parameters and definitions can be found in Table 1 of the “Results” section.

**Table 1:** Sex differences in facial traits of young- and mid-adult Maasai controlled for BMI

Age	Facial trait	Definition	Men		Women		F	Partial Eta^2^	Sig.
			*M*	*SD*	*M*	*SD*			
Young-adults (17–29 years)	Upper fWHR	|Zy-Zy| / |N-Sto|	1.85	0.12	1.85	0.11	0.01	< 0.001	0.955
		|Zy-Zy| / |Gl-Sto|	1.68	0.12	1.67	0.09	0.41	0.004	0.525
		|Zy-Zy| / |N-Ls|	2.15	0.18	2.15	0.14	0.01	< 0.001	0.920
		|Zy-Zy| / |Gl-Ls|	1.93	0.17	1.91	0.12	0.53	0.005	0.470
	Total fWHR	|Zy-Zy| / |N-Gn|	1.16	0.07	1.18	0.06	1.30	0.012	0.257
	Lower fWHR	|Zy-Zy| / |Sn-Gn|	2.05	0.18	2.09	0.16	1.78	0.016	0.185
	Cheekbone prominence	|Zy-Zy| / |Go-Go|	1.25	0.06	1.24	0.05	0.01	< 0.001	0.960
	**Mandibular index**	|Go-Go| / |Sto-Gn|	2.55	0.23	2.63	0.25	3.34	0.030	**0.070**^**+**^
	**Nasal index**	|Al-Al| / |N-Sn|	0.86	0.09	0.81	0.06	9.09	0.078	0.003*
	Mouth shape	|Ls-Li| / |Ch-Ch|	0.45	0.08	0.45	0.06	0.01	< 0.001	0.931
	**Mouth-face index**	|Ch-Ch| / |Zy-Zy|	0.39	0.03	0.38	0.03	4.43	0.038	0.039*
	Height-to-width ratio of the eye (mean)	|Ps-Pi| / |Ex-En|	0.27	0.04	0.28	0.05	1.65	0.015	0.202
Mid-adults (30–50 years)	Upper fWHR	|Zy-Zy| / |N-Sto|	1.78	0.11	1.81	0.11	0.60	0.006	0.441
		|Zy-Zy| / |Gl-Sto|	1.63	0.12	1.64	0.11	0.12	0.001	0.732
		|Zy-Zy| / |N-Ls|	2.02	0.14	2.07	0.15	1.71	0.016	0.194
		|Zy-Zy| / |Gl-Ls|	1.83	0.14	1.84	0.14	0.01	< 0.001	0.905
	Total fWHR	|Zy-Zy| / |N-Gn|	1.15	0.06	1.16	0.06	0.78	0.007	0.378
	Lower fWHR	|Zy-Zy| / |Sn-Gn|	2.08	0.19	2.09	0.20	0.01	< 0.001	0.926
	Cheekbone prominence	|Zy-Zy| / |Go-Go|	1.24	0.05	1.22	0.05	1.61	0.015	0.208
	Mandibular index	|Go-Go| / |Sto-Gn|	2.62	0.30	2.69	0.31	0.77	0.007	0.381
	**Nasal index**	|Al-Al| / |N-Sn|	0.83	0.09	0.81	0.07	3.82	0.034	**0.053**^**+**^
	Mouth shape	|Ls-Li| / |Ch-Ch|	0.39	0.07	0.41	0.08	2.13	0.019	0.148
	Mouth-face index	|Ch-Ch| / |Zy-Zy|	0.40	0.03	0.39	0.03	0.68	0.006	0.412
	Height-to-width ratio of the eye (mean)	|Ps-Pi| / |Ex-En|	0.27	0.06	0.25	0.04	2.71	0.025	0.103

ANCOVA results are presented. Dependent variable: facial trait; independent variables: 1) BMI, 2) sex. Effect size (partial Eta^2^) and significance level (*p*) are presented only for sex (after controlling for BMI). Definitions of the facial landmarks used for facial traits calculation can be found in Fig. 1. Significant sex differences (*) and statistical trends (^+^) are presented in bold

### Anthropometric measurements

Body sexual dimorphism in humans can be studied by a number of parameters that reflect the development of bones, muscles, and adipose tissues. Parameters targeting skeletal development are usually measured in low-fat parts of the human body (e.g., under-chest diameter, wrist and ankle diameters, body height). Muscular development can be assessed by handgrip strength, upper arm, calf, and thigh circumferences, as well as by body mass index (BMI); however, the latter four parameters also account for deposition of fat. In turn, the development of adipose tissues can be measured by various body skinfolds and hips circumference. Combination of different parameters provides more accurate estimates of certain developmental processes.

The data on body height (cm), weight (kg), wrist diameter of the right hand (cm), handgrip strength (kg), triceps skinfold of the right hand (cm), upper arm circumference of the right hand (cm), under-chest, and hips circumferences (cm) were collected. The body height was measured with anthropometer (GPM Swiss made) with an accuracy of ± 0.1 cm. Body weight (mass) was measured by electronic scales (SECA, Germany) accurate to 0.1 kg. BMI was calculated, using bodyweight in kilograms divided by height in meters squared (kg/m^2^). Circumferences were measured by tape with the accuracy of 0.1 cm. Wrist diameter of the right hand was measured by sliding caliper (Martin type, M-222) with the accuracy of 1 mm. Handgrip strength (HGS) was assessed with a portable hand dynamometer (DMER-120, Tulinovsky Instruments, Russia). Participants were instructed to press the dynamometer as hard as they could, in standing position, and with the arm stretched downwards. Right HGS was measured twice and the highest value of the measurements was used in the statistical analyses. Triceps skinfold was measured with baseline skinfold caliper (model 12-110, Lafayette Instrument Company).

### Statistical analysis for classical facial and body measurements

All classical morphological parameters were normally distributed according to Kolmogorov-Smirnov test. Sex differences in BMI were assessed using Student’s *t* test. Sexual dimorphism in classical facial indices was assessed with control for BMI, since this parameter contributed considerably to facial shape variation. For this purpose, analysis of covariance (ANCOVA) was used, where each facial index was set as dependent variable, and BMI and sex, as predictors. To assess the effect size for sex, partial Eta^2^ was used, as it partializes out the effect of other independent variables (in case of multiple predictors), which allows estimating the effect size for each predictor specifically. The associations between facial indices of Maasai men and women with facial centroid size, and body height (as well as a number of other body parameters) were also tested with control for BMI using multivariate analysis of covariance (MANCOVA), where a number of selected traits were set as dependent variables and BMI and a trait of interest (facial centroid size, body height, or any of the other body parameters) served as predictors. The measure of the effect size in these cases was also partial Eta^2^ for the predictor of interest. Association between body height and facial centroid size for men and women was tested using linear regression analysis. For estimating sex differences in body parameters, Student’s *t* test was used. In case of testing subsamples (different age cohorts), the Student’s test was performed with control for equivalence of variances (Levene’s test). Hedges’ g was reported as an effect size for unequal sample sizes. To estimate which body parameters were the main predictors of sex in Maasai, binary logistic regression with stepwise inclusion of predictors (forward Wald algorithm) was used, where sex was set as a dependent variable. To test associations between handgrip strength and body parameters, linear regression (stepwise forward algorithm) was used. Statistical analysis was performed using SPSS version 23.0 (IBM Corp., Armonk, NY, USA). The significance level was set at 0.05.

## Results

### Sexual dimorphism of Maasai facial shape

The degree of sex differences in facial shapes of Maasai were first tested on the general sample including subjects of all ages. Since the age range was very wide (age_min_ = 17; age_max_ = 90), we expected quite noticeable contribution of this factor into the facial shape specificity. Therefore, possible age effects were first eliminated by multivariate analysis of variance where age was set as the first of the two independent variables. According to this linear model, sex (controlled for age) explained only 1.8% of the total variance of the facial shape of Maasai (*p* < 0.001). At the same time, 6% of the total variance in shape was explained by age (*p* < 0.001).

**Fig. 2:**
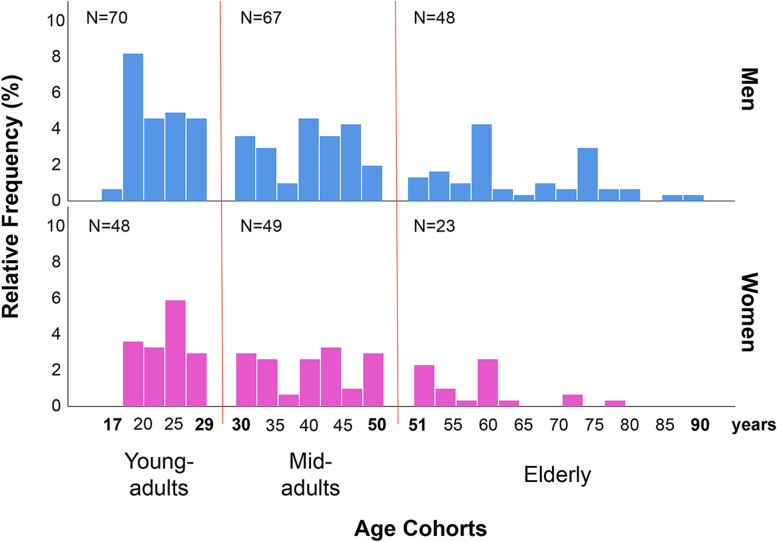
Initial distribution of subjects across age cohorts

Considering very wide age range, there could be some nonlinear associations, since ontogenetic development is not a uniform process, and the intensity and direction of morphogenesis may differ at different stages of individual development. To estimate possible nonlinear effects, we have divided the general sample into three subsamples corresponding to the three age cohorts (young-adults: 17–29 years; mid-adults: 30–50 years; elderly > 50 years). The age thresholds per each cohort were chosen based on the specific features of Maasai social structure (see “Methods” section). This classification has already been implemented in our earlier study of this population [121]. The distribution of subjects across three age cohorts is presented in Fig. 2.

**Fig. 3:**
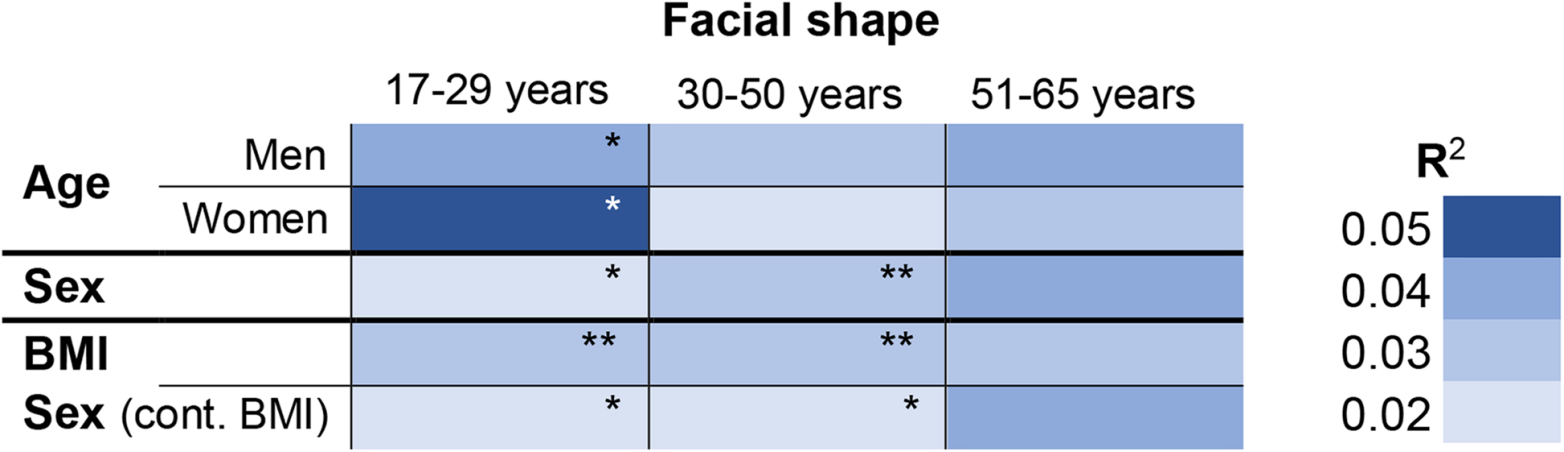
Association between facial shape, age, BMI, and sex in Maasai of different age cohorts. Results of the multivariate ANOVA: facial shape coordinates are dependent variables; **a** age (for men and women was tested separately); **b** sex was set as a single predictor; **c** model with two predictors: BMI and sex. Analysis performed separately per each age cohort: young-adults (17–29 years), mid-adults (30–50 years); elderly (51–65 years). *R*^2^ variance in facial shape explained by independent factor. *p < 0.05, **p < 0.01 (according to permutation test with 10000 permutations)

According to the results, Maasai women demonstrated more pronounced age-related changes in facial shape during young-adult period of life, compared to Maasai men. At the same time, facial shape of Maasai men changed more gradually (Fig. 3a). Generally, sex differences in facial morphology were very weak in the youngest age cohort (17–29 years), but increased with age and were most significant in mid-adults (30–50 years), where sex explained around 3% of variance in facial shape. By the elderly period, sex differences reached the level of 4% of explained variance; however, in the elderly years, differences were not statistically significant (Fig. 3b).

**Fig. 4:**
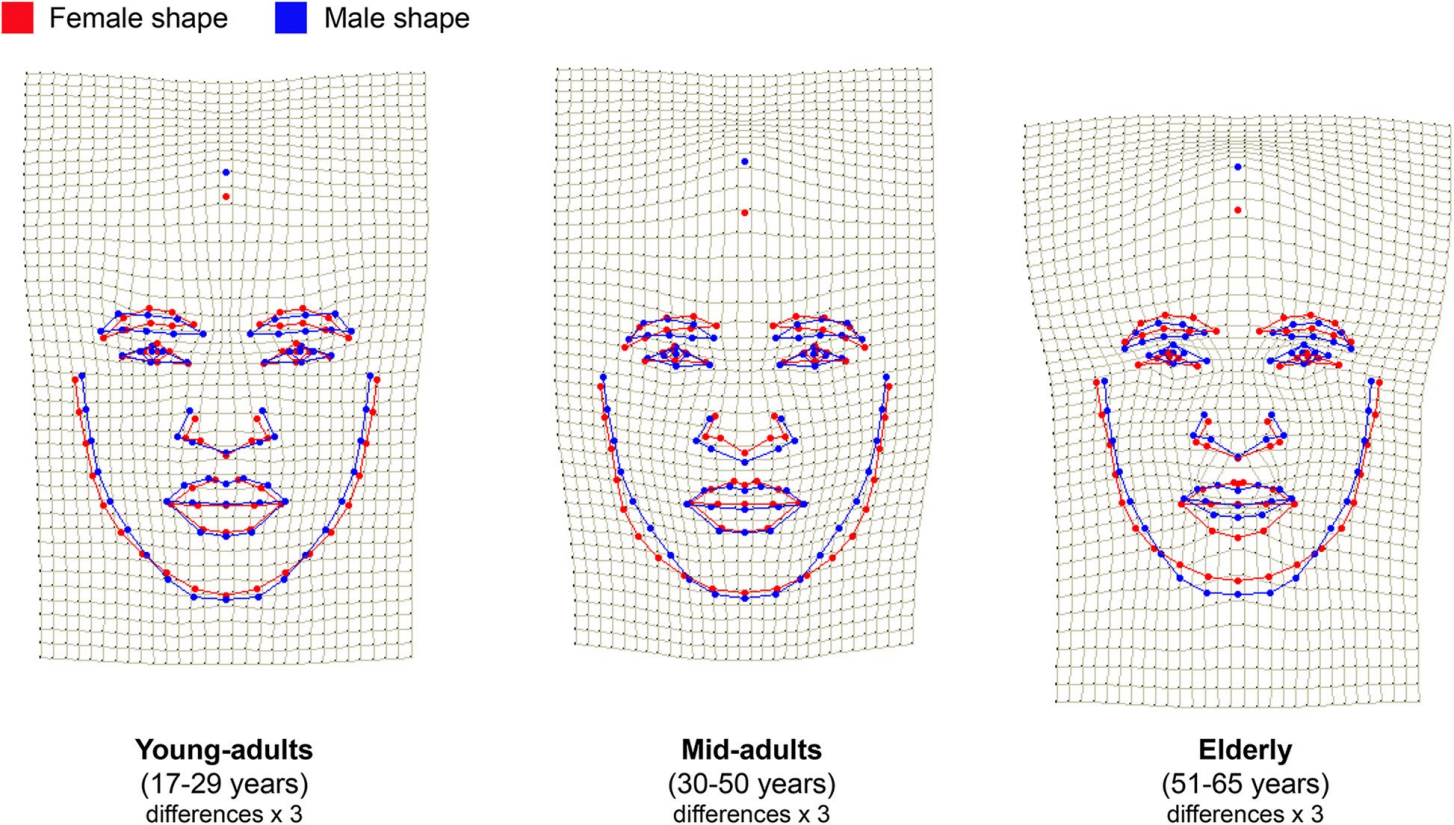
Sex differences in facial shape of Maasai from three age cohorts. Sexual dimorphism in facial shape of Maasai from three age cohorts: young-adults (*N* = 118; var. expl. 2%; *p* = 0.043); mid-adults (*N* = 116; var. expl. 3%; *p* = 0.003); elderly (*N* = 71; var. expl. 4%; *p* = 0.113). Mean female shape per each age group (in red) is a reference configuration, the thin-plate grid is deformed toward male shape (in blue) with an exaggeration by a factor of 3

Next, we have tested the impact of BMI on the facial shape in each of the age cohorts. Sex differences in BMI were significant for young-adults (mean BMI_women_ = 21.0 ± 3.8; mean BMI_men_ = 19.8 ± 2.2; Student’s *t* test: *N* = 111; *t* = − 2.161; *p* = 0.033), and mid-adults (mean BMI_women_ = 21.7 ± 3.5; mean BMI_men_ = 19.8 ± 2.3; Student’s *t* test: *N* = 110; *t* = − 3.293; *p* = 0.001), with women having higher BMI than men, which was especially pronounced in mid-adult cohort. The BMI was significantly associated with facial shape both in young- and mid-adult Maasai. Results of the analysis revealed that increase in sexual dimorphism in facial shape within the mid-adult cohort was at least partially caused by the BMI differences between men and women, since after controlling for BMI (by adding this parameter as the first of the two independent variables in the multivariate analysis of variance), sexual dimorphism in Maasai facial shape within the mid-adult cohort decreased (Fig. 3c). The latter indicates that within the whole age range of 17–50 years, sex explains about 2% of the total variance of the facial shape of Maasai. When young-adults and mid-adults samples were compared, it was founds that in age cohort between 30 and 50 years, differences in BMI between men and women added to increase of sexual dimorphism in facial shape. This is well illustrated by the visualization of the sex differences in facial shape for each age cohort presented in Fig. 4 (the thin-plate grid was deformed from the reference shape (female, in red) toward the male shape (in blue); for enhancing the details, the differences were exaggerated by a factor of 3).

The visualizations presented in Fig. 4 support statistical results. In the youngest age cohort, sex differences in facial shape were very small—even after 3-fold exaggeration, male and female mean configurations almost coincided. However, even here the tendency for narrower and more prolonged in vertical plane male faces is recognizable. Among young-adults, men also had slightly wider noses and narrower-set and lower eyebrows, as well as higher forehead hairline. Generally, this tendency reached its’ peak in mid-adult cohort, when female faces were relatively wider, especially in the area of bigonial width, and female nasal shape reflected generally smaller noses in both width and height directions. However, wider female faces compared to males’, partly represent higher female BMI, which may result in increased fat deposition in the cheeks area. In the elderly age cohort, the general degradation of the lower lip vermillion is observed in males, as well as further narrowing of the lower face and vanishing of the sex-specific male morphology in the eyebrows and nasal regions of the face. At the same time, female facial traits within the elderly cohort changed less dramatically compared to younger ages, which may suggest that aging processes affected male facial morphology to a greater extent.

**Fig. 5:**
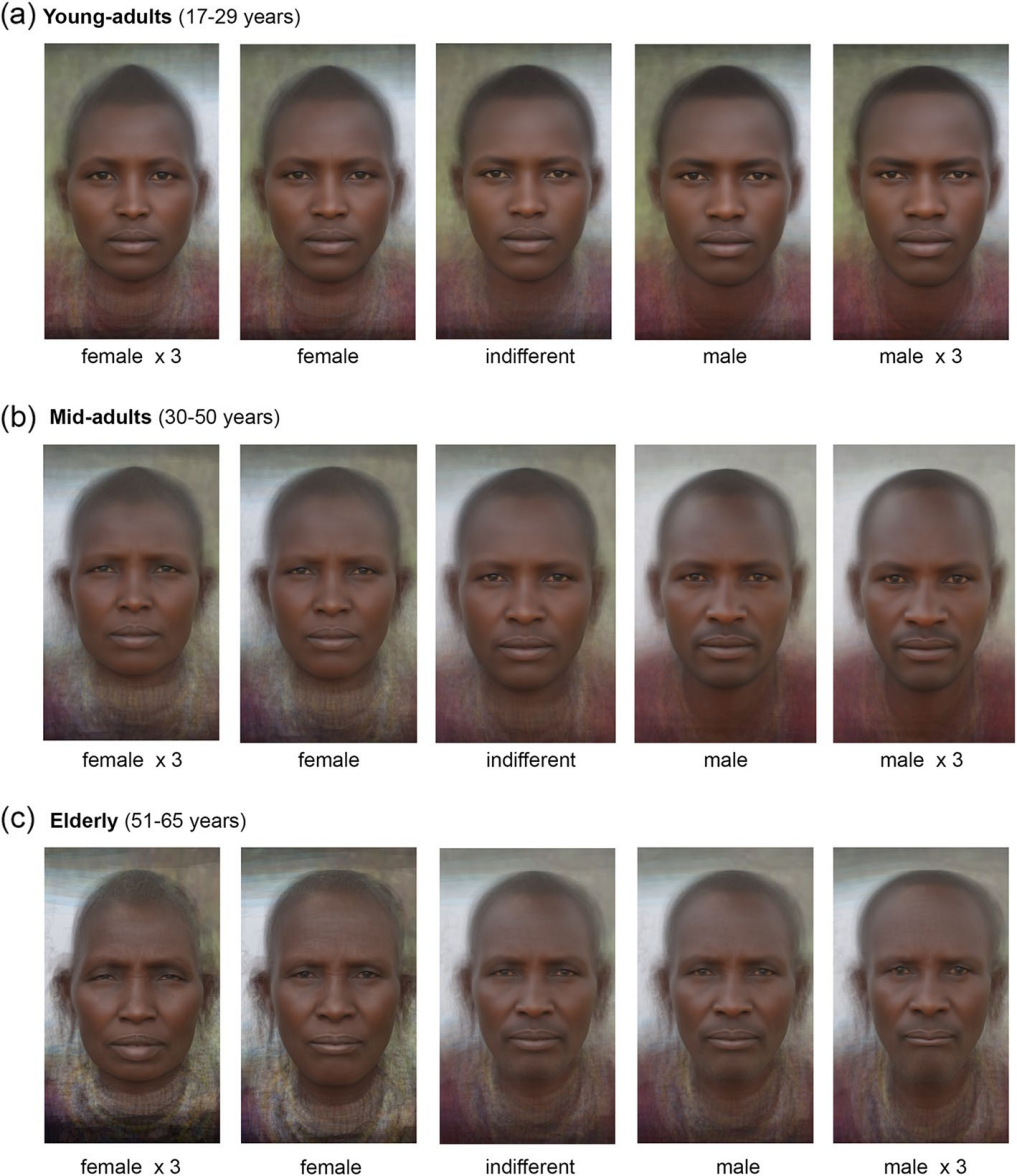
Averaged facial portraits representing differences between Maasai men and women. Sex differences in facial shape of Maasai from three age cohorts. Geometric morphometric morphs display the facial shape change from a sexually undifferentiated face (in the middle) toward the average female (to the left) and male (to the right) face. The outer configurations represent differences exaggerated by a factor of 3. Sex differences were significant for young-adults (var. expl. = 2%, *p* < 0.05), and for mid-adults (var. expl. = 3%, *p* < 0.01) (according to permutation test with 10000 permutations)

For clarity, Fig. 5 represents facial shape differences between Maasai men and women as averaged individual portraits unwarped upon target configurations across three age cohorts.

### Sexual dimorphism in facial indices of adult Maasai

Despite the fact that some significant sex differences in Maasai facial shapes could be visualized after 3-fold exaggeration, generally, male and female Maasai faces were very similar. Analysis of sex differences in a number of facial indices, which are usually considered sexually dimorphic, are presented in Table 1. Analysis (with control for BMI) was performed only for young- and mid-adults, since only in these age cohorts significant sex differences in facial shape were revealed by geometric morphometrics. Upper facial width-to-height ratio (fWHR) was calculated in all four known variants, measured both from the eyebrows (*glabella*, Gl) and upper eyelids (*nasion*, N) to the upper lip line (*labiale superius*, Ls) and the line between upper and lower lips vermillions (*stomion*, Sto) [37]. Since lip vermillions in Maasai are very thick, as well as in most of African and some other populations, *stomion* approximation in this case is more informative [33, 37, 76].

As was expected based on geometric morphometrics, none of the variants of the upper fWHR was sexually dimorphic in Maasai; moreover, women on average had slightly higher values of this trait (although differences were not significant). The most sexually dimorphic regions of the face were the shape of the jaw (mandible index, marginally significant in young-adults), nose (nasal index in young-adults and marginally significant in mid-adults), and mouth (mouth-face index in young-adults) (Table 1).

### Sexual dimorphism in Maasai body parameters

Mean values of tested body parameters and significance of sex differences are presented in Table 2.

**Table 2:** Sex differences in body parameters of young- and mid-adult Maasai

Age	Body parameter	Unit	Men		Women		***t***(df)	Hedges’ g	Sig.
			*M*	*SD*	*M*	*SD*			
Young-adults (17–29 years)	Body height	cm	167.5	7.1	155.3	5.4	10.02(115)	1.88	< 0.001
	Body weight	kg	55.6	8.5	50.7	9.5	2.87(110)	0.55	0.005
	Body mass index (BMI)	kg/m^2^	19.8	2.2	21.0	3.8	− 2.16(109)	− 0.41	0.033
	Handgrip strength	kg	38.4	8.9	26.2	4.7	9.62(110)^a^	1.63	< 0.001
	Wrist diameter	mm	55.9	3.6	52.0	5.1	4.83(109)	0.92	< 0.001
	Upper arm circumference	cm	24.4	2.4	24.2	2.5	0.43(114)	−	0.668
	Triceps skinfold	mm	8.7	5.4	13.8	5.4	− 4.97(115)	− 0.94	< 0.001
	Under-chest circumference	cm	77.5	4.7	72.8	4.9	5.32(115)	0.98	< 0.001
	Hips circumference	cm	87.4	6.3	90.6	5.7	− 2.76(112)	− 0.53	0.007
	Facial centroid size	cm	56.0	3.8	51.6	3.4	6.35(116)	1.20	< 0.001
Mid-adults (30–50 years)	Body height	cm	168.1	6.4	156.3	6.1	9.89(114)	1.88	< 0.001
	Body weight	kg	56.1	8.7	53.1	9.1	1.81(108)	0.33	0.073
	Body mass index (BMI)	kg/m^2^	19.8	2.4	21.7	3.5	− 3.14(79)^a^	− 0.65	0.001
	Handgrip strength	kg	37.9	7.9	25.9	5.4	9.14(112)	1.72	< 0.001
	Wrist diameter	mm	56.1	3.7	53.2	5.5	3.26(109)	0.64	0.002
	Upper arm circumference	cm	24.9	2.3	27.2	9.2	− 1.90(74)^a^	− 0.37	0.087
	Triceps skinfold	mm	9.0	8.3	16.6	7.5	− 5.10(114)	− 0.95	< 0.001
	Under-chest circumference	cm	79.9	5.8	74.7	7.8	4.17(114)	0.77	< 0.001
	Hips circumference	cm	89.4	6.3	93.8	7.5	− 3.38(112)	− 0.65	0.001
	Facial centroid size	cm	57.9	3.8	53.1	3.4	6.99(114)	1.32	< 0.001

Results of the Student’s *t* test with control for equivalence of variances (Levene’s test) are presented; df – degrees of freedom

^a^Adjusted according to significant results of the Levene’s test. Hedges’ g effect size for unequal sample sizes (negative when female values are larger than male)

With an exception of body weight in older age cohort, all body parameters were sexually dimorphic, with highest level of dimorphism (more than 1 SD) for body height, handgrip strength, and facial size both for young and mid-adults.

**Fig. 6:**
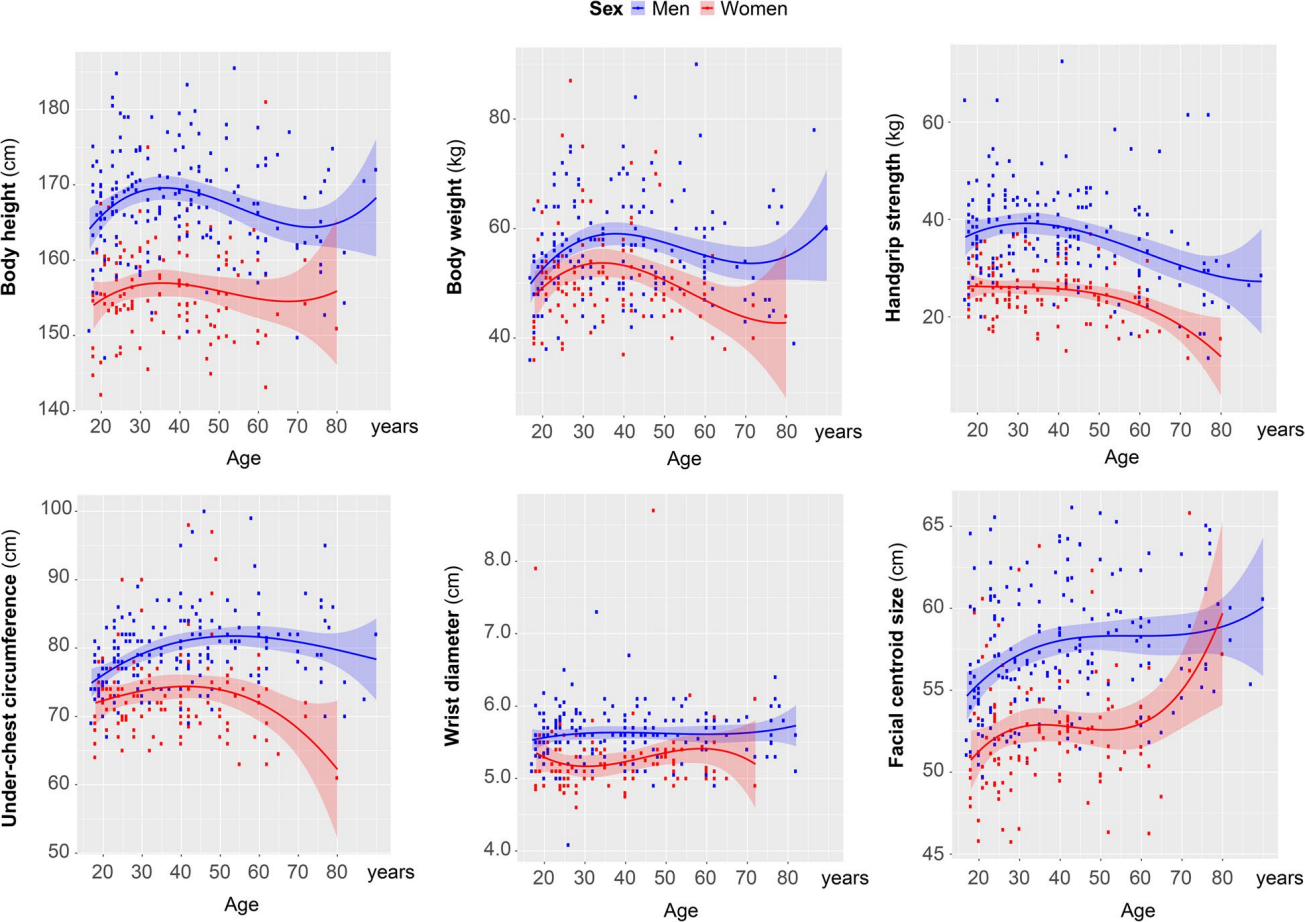
Male-specific body parameters. Results of the regression analysis per each parameter: body height: cubic model (men: *R*^2^ = 0.065, *p* = 0.007; women: *R*^2^ = 0.019, *p* = 0.525); body weight: cubic model (men: *R*^2^ = 0.079, *p* = 0.003; women: *R*^2^ = 0.080, *p* = 0.026); handgrip strength: cubic model (men: *R*^2^ = 0.105, *p* < 0.001; women: *R*^2^ = 0.146, *p* < 0.001); under-chest circumference: cubic model (men: *R*^2^ = 0.148, *p* < 0.001; women: *R*^2^ = 0.059, *p* = 0.071); wrist diameter: cubic model (men: *R*^2^ = 0.009, *p* = 650; women: *R*^2^ = 0.027, *p* = 0.405); facial centroid size (men: *R*^2^ = 0.107, *p* < 0.001; women: *R*^2^ = 0.074, *p* = 0.029)

**Fig. 7:**
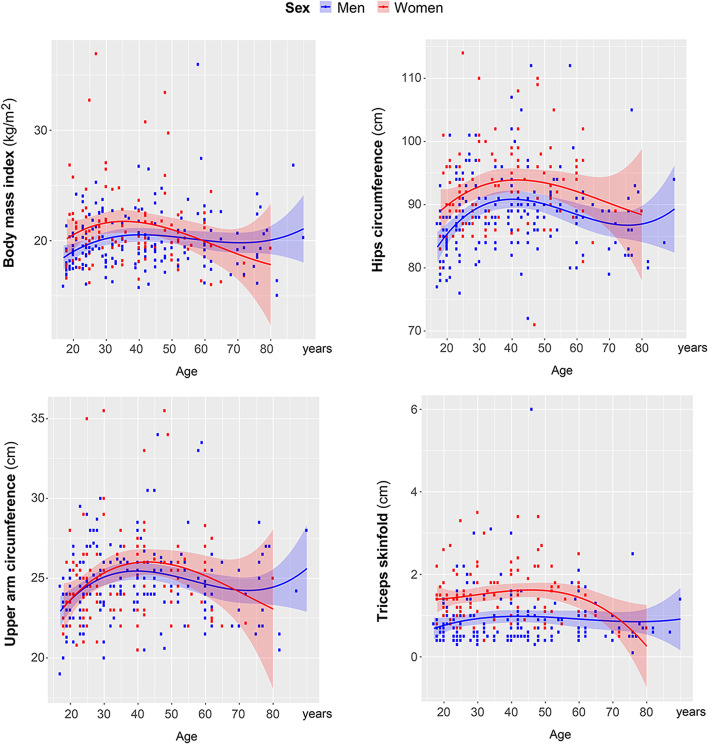
Female-specific body parameters. Results of the regression analysis per each parameter: body mass index: cubic model (men: *R*^2^ = 0.046, *p* = 0.043; women: *R*^2^ = 0.044, *p* = 0.173); hips circumference: cubic model (men: *R*^2^ = 0.112, *p* < 0.001; women: *R*^2^ = 0.051, *p* = 0.024); upper arm circumference: cubic model (men: *R*^2^ = 0.082, *p* = 0.002; women: *R*^2^ = 0.050, *p* = 0.120); triceps skinfold: cubic model (men: *R*^2^ = 0.016, *p* = 0.406; women: *R*^2^ = 0.063, *p* = 0.056)

On the next step, we tested the distribution of the body traits’ values across all ages for men and women. These distributions were best explained by nonlinear regression models. Some of the body parameters had generally higher values in men (body height, body weight, handgrip strength, under-chest circumference, wrist diameter, facial centroid size; Fig. 6), while others were more characteristic of women (higher body mass index, higher hips and upper arm circumferences, and larger triceps skinfold; Fig. 7). Male-specific parameters were more related to general body size, massiveness, and strength, while female-specific parameters were mostly related to increased body fat deposition, which is generally more typical for women than men.

Our cross-sectional data demonstrate that most of the body parameters had non-linear developmental trajectory in men and women during the lifespan (Figs. 6 and 7). Especially, this was the case for those parameters, which were initially better developed in women than in men (Fig. 7). Female BMI, hips circumference, and upper arm circumference along with triceps skinfold exceeded those of men in adulthood, but crucially decreased closer to elderly years of Maasai women. Taking into consideration such sex-specific nonlinear changes in body parameters of Maasai, only young- and mid-adult age cohorts were addressed for testing general sex differences in body parameters.

It can be already deduced from Figs. 6 and 7 which body measures are higher in Maasai men, and which in women. However, to see which body parameters contribute the most to the body sexual dimorphism, we have run stepwise binary logistic regression with forward Wald algorithm. The following independent variables were entered to the analysis: body height, body weight, handgrip strength, under-chest circumference, wrist diameter, facial centroid size, BMI, hips circumference, upper arm circumference, and triceps skinfold. Independent variables were added to the model stepwise starting from the first predictor with the most powerful predicting power. Each next variable is added on the consequent step improving the model until its quality drops. Those predictors, which do not add to the model capacity, were not included. Here, stepwise regression was terminated at the step 4, and hence the model included 4 main predictors of sex (namely body height, under-chest and hips circumferences, and handgrip strength). The results of the regression are presented in Table 3. The model based on these four body parameters was able to predict Maasai sex with probability of 88%.

**Table 3:** Body sexual dimorphism in young- and mid-adult Maasai

Dependent variable: sex (female)				
*Predictors*	***B***	***P***	p(model)	***R***^2^
Body height	− 17.311	0.001	< 0.001	0.881
Under-chest circumference	− 0.336	< 0.001		
Handgrip strength	− 0.234	0.001		
Hips circumference	0.484	< 0.001		

Binary logistic regression (stepwise, forward Wald algorithm). Independent variables, which entered the analysis: body height, body weight, BMI, handgrip strength, wrist diameter, upper arm circumference, triceps skinfold, under-chest circumference, hips circumference, facial centroid size

### Facial parameters and allometry in Maasai

To test for allometric effects on facial traits of young- and mid-adult Maasai, we have analyzed possible associations of the male and female facial shapes with body height, and facial centroid size (taken as natural logarithm of centroid size), as two main measures of static allometry in humans [46]. Since it was already demonstrated that faces of young (17–29 years) and mid-adult (30–50 years) Maasai had similar patterns of sexual variability, with BMI being the main distinguishing factor (Figs. 3 and 4), these two age cohorts were pooled for static allometry testing. The Procrustes superimposition with sliding semilandmarks, and subsequent symmetrization, were held separately for men and women from 17 to 50 years age cohort. Geometric morphometric analysis revealed no considerable association of facial shape with body height either in men (*N* = 135, var. expl. = 1%, *p* = 0.075), or women (*N* = 96, var. expl. = 2%, *p* = 0.092). At the same time, facial shape was significantly associated with facial centroid size in both sexes (men: *N* = 136, var. expl. = 6%, *p* < 0.001; women: *N* = 96, var. expl. = 7%, *p* < 0.001). Even after controlling for BMI, this association remained highly significant (men: *N* = 128, var. expl. = 6%, *p* < 0.001; women: *N* = 91, var. expl. = 6%, *p* < 0.001).

To assess quantitatively, which facial areas were most sensitive to allometric effects, we used multivariate analysis of covariance (MANCOVA), where the set of facial parameters (upper fWHR in four variants, total fWHR, lower fWHR, cheekbone prominence, mandibular index, nasal index, mouth shape, mouth-face index, height-to-width ratio of the eye) was assigned as dependent variables, and BMI and facial centroid size (CS) (or alternatively body height) were set as covariates. Results revealed no associations between facial parameters and body height for men. For women, there were very weak relation between body height and upper, total, and lower fWHR, with partial Eta^2^ not exceeding the level of 0.06; significance level for these parameters could not survive Bonferroni correction for testing 12 variables. Details can be found in Supplementary Table 1. Worth noting that without BMI control, facial parameters also were not related to body height (Suppl. Tab. 2).

**Fig. 8:**
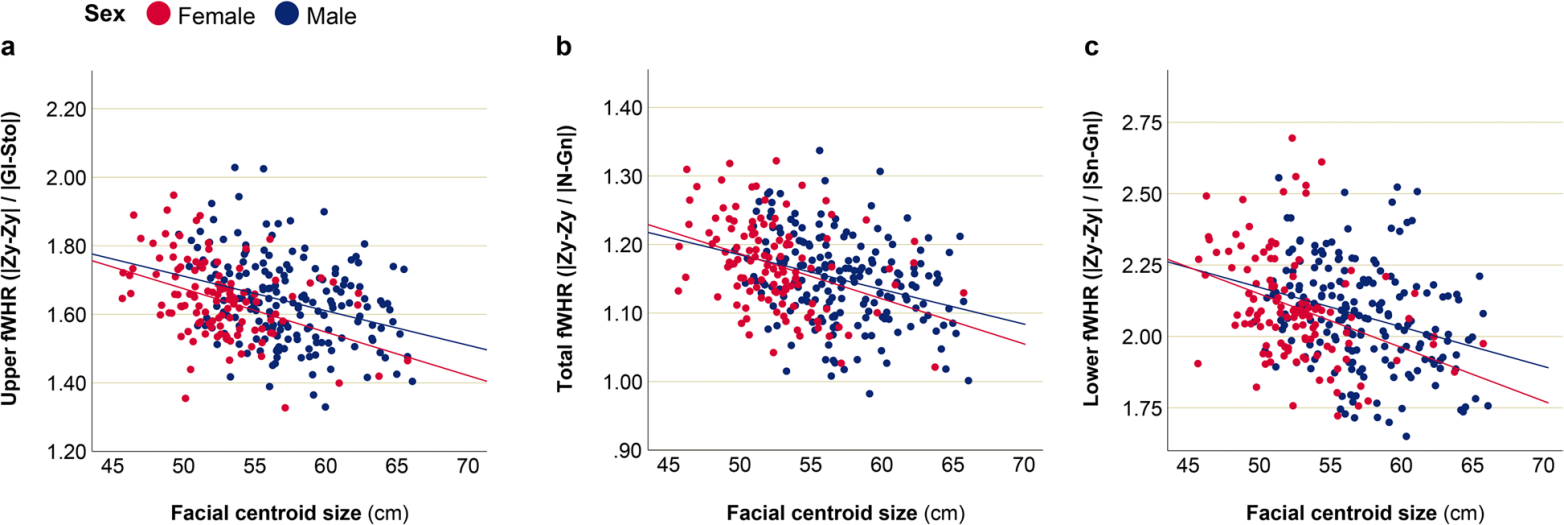
Association between fWHRs and facial centroid size in Maasai men and women (controlled for BMI). **a** men: *R*^2^ = 0.095, women: *R*^2^ = 0.164; **b** men: *R*^2^ = 0.090, women: *R*^2^ = 0.136; **c** men: *R*^2^ = 0.085, women: *R*^2^ = 0.132

However, a number of associations were revealed between facial indices and size of the face (CS) (Table 4, Fig. 8). Both in Maasai men and women, upper, total, and lower fWHR were significantly associated with facial centroid size, with larger faces having lower values of these parameters. Thus, larger faces in Maasai were characterized by narrower and prolonged in vertical direction shape.

**Table 4:** Association between facial traits and facial centroid size in Maasai (controlled for BMI)

Covariate	Dependent variables	Definition	***F***	Partial Eta^**2**^	Sig.
**Men**					
Facial CS	**Upper fWHR**	|Zy-Zy| / |N-Sto|	6.09	0.046	0.015
		**|Zy-Zy| / |Gl-Sto|**	16.84	0.117	< 0.001*
		|Zy-Zy| / |N-Ls|	5.90	0.045	0.017
		**|Zy-Zy| / |Gl-Ls|**	16.34	0.114	< 0.001*
	**Total fWHR**	|Zy-Zy| / |N-Gn|	22.3	0.150	< 0.001*
	**Lower fWHR**	|Zy-Zy| / |Sn-Gn|	19.1	0.132	< 0.001*
	Cheekbone prominence	|Zy-Zy| / |Go-Go|	5.61	0.039	0.025
	Mandibular index	|Go-Go| / |Sto-Gn|	7.01	0.054	0.008
	Nasal index	|Al-Al| / |N-Sn|	0.88	0.007	0.350
	Mouth shape	|Ls-Li| / |Ch-Ch|	3.61	0.028	0.060
	Mouth-face index	|Ch-Ch| / |Zy-Zy|	3.95	0.030	0.050
	Height-to-width ratio of the eye (mean)	|Ps-Pi| / |Ex-En|	3.02	0.023	0.084
**Women**					
Facial CS	**Upper fWHR**	|Zy-Zy| / |N-Sto|	13.03	0.128	0.001*
		|Zy-Zy| / |Gl-Sto|	20.78	0.189	< 0.001*
		|Zy-Zy| / |N-Ls|	11.25	0.112	0.001*
		|Zy-Zy| / |Gl-Ls|	19.92	0.183	< 0.001*
	**Total fWHR**	|Zy-Zy| / |N-Gn|	18.72	0.174	< 0.001*
	**Lower fWHR**	|Zy-Zy| / |Sn-Gn|	11.96	0.119	0.001*
	Cheekbone prominence	|Zy-Zy| / |Go-Go|	0.60	0.006	0.452
	Mandibular index	|Go-Go| / |Sto-Gn|	6.35	0.066	0.014
	Nasal index	|Al-Al| / |N-Sn|	0.46	0.005	0.500
	Mouth shape	|Ls-Li| / |Ch-Ch|	0.70	0.008	0.405
	Mouth-face index	|Ch-Ch| / |Zy-Zy|	0.26	0.003	0.613
	Height-to-width ratio of the eye (mean)	|Ps-Pi| / |Ex-En|	0.47	0.005	0.495

MANCOVA results are presented. Dependent variables: facial traits; independent variables: 1) BMI, 2) facial centroid size (CS). Effect size (partial Eta^2^) and significance level (*p*) are presented only for CS (after controlling for BMI). Definitions of the facial landmarks used for facial traits calculation can be found in Fig. 1. Significant associations, which survived Bonferroni correction for testing 12 variables are marked with *, and presented in bold

These results suggest that body height and facial size are not directly associated in Maasai (especially in men), and allometric effect on facial traits occurs locally: face growth results in the narrower and prolonged in vertical direction faces, but is not strongly related to body growth. Analysis of association between body height and facial CS confirmed that such tendency was especially pronounced in men: body height explained only around 4% of variance in male facial centroid size (linear regression: Beta = 0.207, *R*^2^ = 0.043, *p* = 0.016), whereas for women this relation was stronger (Beta = 0.324, *R*^2^ = 0.105, *p* = 0.001).

### Association between facial and body parameters

The association between facial and body parameters were also tested pooling young- and mid-adult cohorts (age range 17–50 years), with analysis performed controlling for BMI. For this purpose, MANCOVA was applied, where the full set of 12 facial indices was assigned as dependent variables, and BMI and each of the body measures were set as covariates. The list of body parameters used for association with facial traits was restricted to those, which represent skeletal and muscular development (wrist diameter, upper arm circumference, under-chest circumference, hip circumference), rather than fat deposition. Association between facial shape and handgrip strength in the same Maasai sample has been previously demonstrated and discussed in detail in other our publication [120]; therefore, we do not directly focus on this parameter here, but rather estimate it through other body traits, which were not tested before. Analysis was held separately for men and women.

**Fig. 9:**
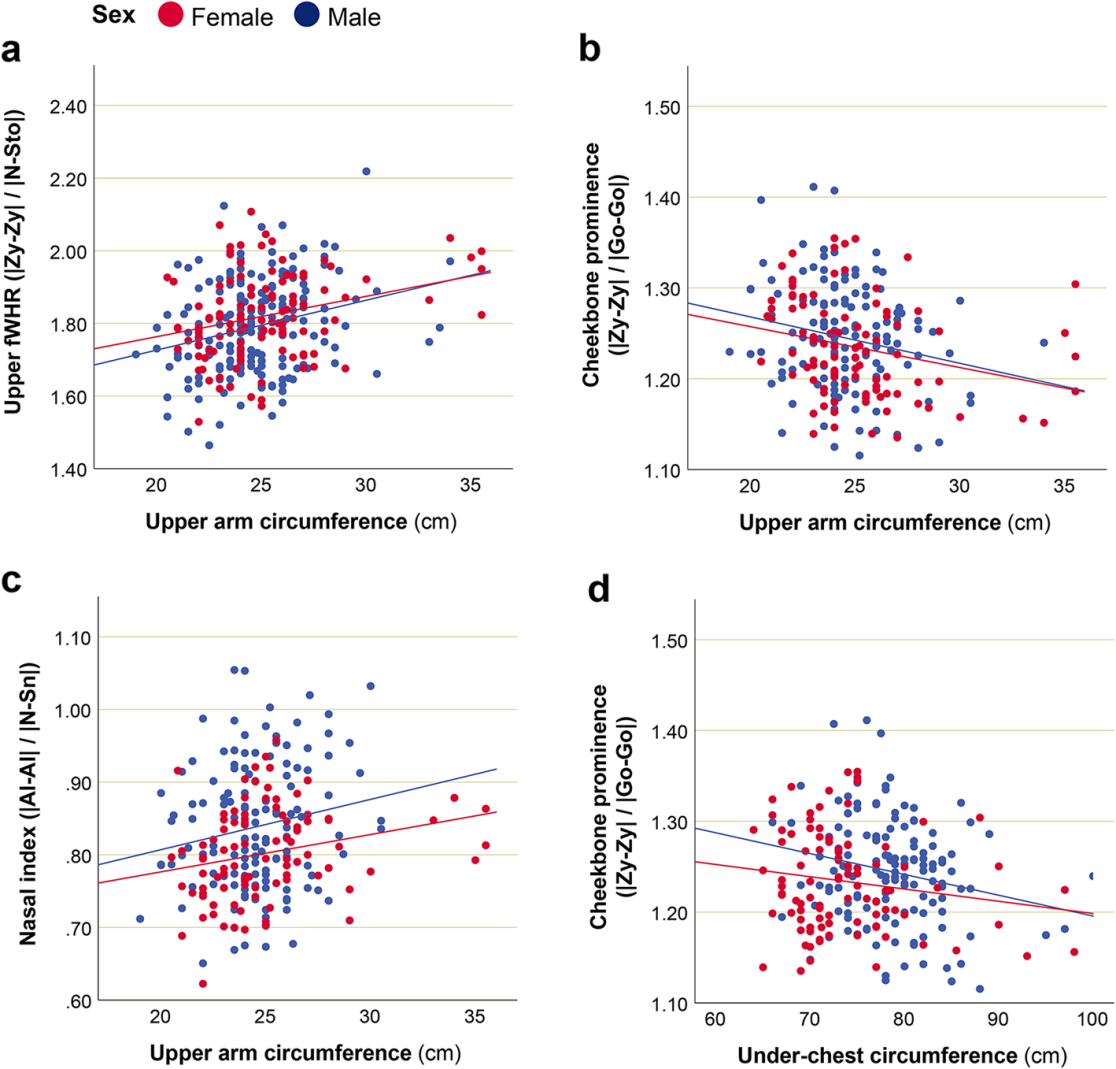
Association between facial traits and body parameters in young- and mid-adult Maasai. **a** Men: *R*^2^ = 0.076, women: *R*^2^ = 0.072; **b** men: *R*^2^ = 0.042, women: *R*^2^ = 0.069; **c** men: *R*^2^ = 0.034, women: *R*^2^ = 0.067; **d** men: *R*^2^ = 0.045, women: *R*^2^ = 0.026

The results revealed associations between upper arm circumference, upper fWHR, and cheekbone prominence for men, as well as upper arm circumference and nasal index for women. The under-chest circumference was also associated with cheekbone prominence in men. All these associations stood significant after Bonferroni correction for testing 12 variables (Table 5; Fig. 9). Details on all parameters can be found in Supplementary Table 3.

**Table 5:** Association between facial traits and body parameters in Maasai (controlled for BMI)

Predictor	Dependent variables	Definition	***F***	Partial Eta^**2**^	Sig.
**Men**					
Upper arm circumference	Upper fWHR	|Zy-Zy| / |N-Sto|	10.20	0.075	0.002
	Cheekbone prominence	|Zy-Zy| / |Go-Go|	9.02	0.067	0.003
Under-chest circumference	Cheekbone prominence	|Zy-Zy| / |Go-Go|	9.90	0.073	0.002
**Women**					
Upper arm circumference	Nasal index	|Al-Al| / |N-Sn|	11.84	0.119	0.001

Only significant results of MANCOVA are presented. Dependent variables: facial traits; independent variables: 1) BMI, 2) each of body parameters. Effect size (partial Eta^2^) and significance level (*p*) are presented only for each body parameter (after controlling for BMI). Definitions of the facial landmarks used for facial traits calculation can be found in Fig. 1. All presented associations stood significant after Bonferroni correction for testing 12 variables. Full results can be found in Supplementary Tab. 3

According to obtained results, larger upper arm circumference of the right hand was significantly associated with higher upper fWHR (measured as |Zy-Zy| / |N-Sto|) and relatively higher bigonial width (i.e., lower cheekbone prominence measured as |Zy-Zy| / |Go-Go|) in Maasai men. For women, this relation was not significant. Relatively higher bigonial width was also detected for men with larger under-chest circumference. This relation was also not statistically significant for women. However, upper arm circumference was positively related to relative nasal width (nasal index) in Maasai women, whereas for men this association was not significant.

In our earlier study, it was demonstrated that physical strength of Maasai was positively associated with facial width, both in zygomatic and bigonial areas [121]. We proposed that upper arm and under-chest circumferences could be viewed as parameters related to male muscular and skeletal development, and thus could be associated with physical strength. To test this assumption, we analyzed relationship between handgrip strength and body parameters using linear regression with stepwise (forward) inclusion of predictors. The analysis was held for men and women separately, and the list of predictors included body height, body weight, BMI, wrist diameter, upper arm circumference, triceps skinfold, under-chest circumference, hips circumference, and facial centroid size. Regression model for men was terminated at step 3, resulting in 3 predictors (upper arm circumference, wrist circumference, and triceps skinfold), which together explained around 27% of variance in handgrip strength (*p* < 0.001) (Table 6). For women, no significant associations were revealed.

**Table 6:** Association between handgrip strength and body parameters in Maasai men

Dependent variable: handgrip strength in men				
*Predictors*	***B***	***P***	p(model)	***R***^**2**^
Upper arm circumference	1.697	< 0.001	< 0.001	0.268
Wrist circumference	0.564	0.002		
Triceps skinfold	− 0.359	0.022		

Linear regression with stepwise inclusion of predictors (forward algorithm). Independent variables, which entered the analysis: body height, body weight, BMI, wrist diameter, upper arm circumference, triceps skinfold, under-chest circumference, hips circumference, facial centroid size

Thus, according to the results, higher handgrip strength in men was positively associated with upper arm and wrist circumferences, and negatively with triceps skinfold. This, at least partially, corresponds to our expectations, confirming that more physically developed (and strong) Maasai men had relatively wider faces, both in mid- and lower facial areas. Although, these traits were not generally sex-specific (Table 1).

## Discussion

In this study, it was demonstrated that sexual facial dimorphism in Maasai is rather low, compared to Europeans and Asians [38, 124], and sex explains 1.8% of the total variance of facial shape in the whole sample (for instance, in Buryats, a Mongolian population of Southern Siberia, sex explains 20% of the total facial shape variance, which was demonstrated using the same configuration of facial landmarks as in the present study [124]). When the three age cohorts of Maasai were tested separately, it was found that sex differences in facial morphology were minimal (2%) in the youngest age cohort, and increase with age, gaining up to 4% in in elder-adults age cohort. However, after controlling for BMI, sexual dimorphism in Maasai facial shape within the mid-adult cohort decreased. Despite the small dimorphism, the morphometric data pointed to relatively narrower and vertically prolonged faces, slightly wider noses, narrower-set and lower eyebrows, slightly wider mouth, and higher forehead hairline in males. Female faces were wider, especially in the aria of bigonial width, shorter and rounded, their noses were smaller both in width and height, and these differences were maximal in mid-age cohort. As for the more rounded faces and smaller noses, the Maasai women were similar to women from Caucasian origin from Slovenia [134]. Both in Maasai and Caucasian sample, the gender-dependent characteristics were even more pronounced in the middle-age and older adults [134]. The data on sexual dimorphism in Maasai faces are in certain contradiction to previous findings from Caucasian populations, for which significant sex dimorphism in facial shape was reported [134, 135]. Maasai women have wider faces compared to men, and this tendency remained visible with control for BMI. Similar sexual differences were reported by us earlier in Buryats, the Mongolian origin people from Southern Siberia [37]. On the contrary, in Caucasian and majority of studied Asian populations, men usually have relatively wider faces compared to women [33, 70] (see review on 32 populations: [67]).

Men and women Maasai demonstrate age-related differences in changes of facial shape. Such changes were especially evident in women between 17 and 29 years of age. Our data suggest that, at least to some degree, the increase of facial sex dimorphism with age may be due to the effect of BMI (higher in women, compared to men, as well as increasing in women with age). Aging processes in older age-cohort affected male facial morphology more compared to women. Men demonstrate the degradation of the lower lip vermillion, further narrowing of the lower face and decrease of the sex-specific male morphology in the eyebrows and nasal regions. We conclude that the most sexually dimorphic regions of the face in Maasai were the lower jaw and the nose.

One of the goals of our study was to address the debates on sex-typical variation in fWHR and its’ evolutionary origin. Recently published paper by Hodges-Simeon with co-authors [72] based on data from Caucasian origin (from 3 to 40 years) and Bolivian Tsimane (from 7 to 21 years) samples of men and women tested the applicability of four variants of fWHR measurements and demonstrated that fWHR lower (including lower jaw) exhibited both adult sex differences, and the classic pattern of ontogeny for human secondary sexual characteristics (greater lower-face growth in male adolescents relative to females). In our study, we calculated six variants of fWHR (including four variants of upper fWHR, total, and lower fWHR), but none of them revealed significant sexual dimorphism. The Maasai women on average, with control for BMI, had slightly higher (although non-significant) values of upper fWHR. Taking the data from other populations [38, 75–0], we conclude that sexual dimorphism in upper fWHR may not be universal for humans. Few years earlier, Kramer has arrived to identical conclusion, on the basis of meta-analysis of human skull [71]. And given resent data from relatively big sample of 131 chimpanzees, represented three subspecies (*Pan troglodytes verus*, *P. t. schweinfurthii*, *P. t. troglodytes*), fWHR may not be considered as sex-related trait in this species either [97].

To what extent facial dimorphism, particularly masculinity traits, may be related to reproduction in Maasai population remained to be tested. However, the data from two traditional non-western societies—the Agta of Philippines, egalitarian forest nomadic hunter gatherers, practicing monogamous marriages, bilocal and exogamic, and monogamous Maya of western Belize, practicing mixture of slash-and-burn agriculture and paid labour, with high rate of arranged marriages, and patrilocal or neolocal residence—provide no evidence that offspring of males with higher facial masculinity survive better [136]. Recent meta-analysis testing relationships between sexually dimorphic traits and reproduction in men revealed no association between facial masculinity and either mating or reproduction [29, 137].

Our findings on low level of facial shape sexual dimorphism are in total agreement with findings reported by other scholars for other African populations, particularly Namibian Nama and Cameroonian Bantu [38]. The lack of sexual dimorphism in shape may be compensated by sex differences in color, preferences for lighter skin in females were found in some African populations [138–140]. Besides, in Cameroonian females, the skin lightness was positively correlated with perceived femininity. Skin lightness in the Cameroonian, Iranian, and Turkish male faces was negatively correlated with masculinity [141]. Hence, in non-European populations, the facial shape dimorphism may be in certain association with skin color.

**Fig. 10:**
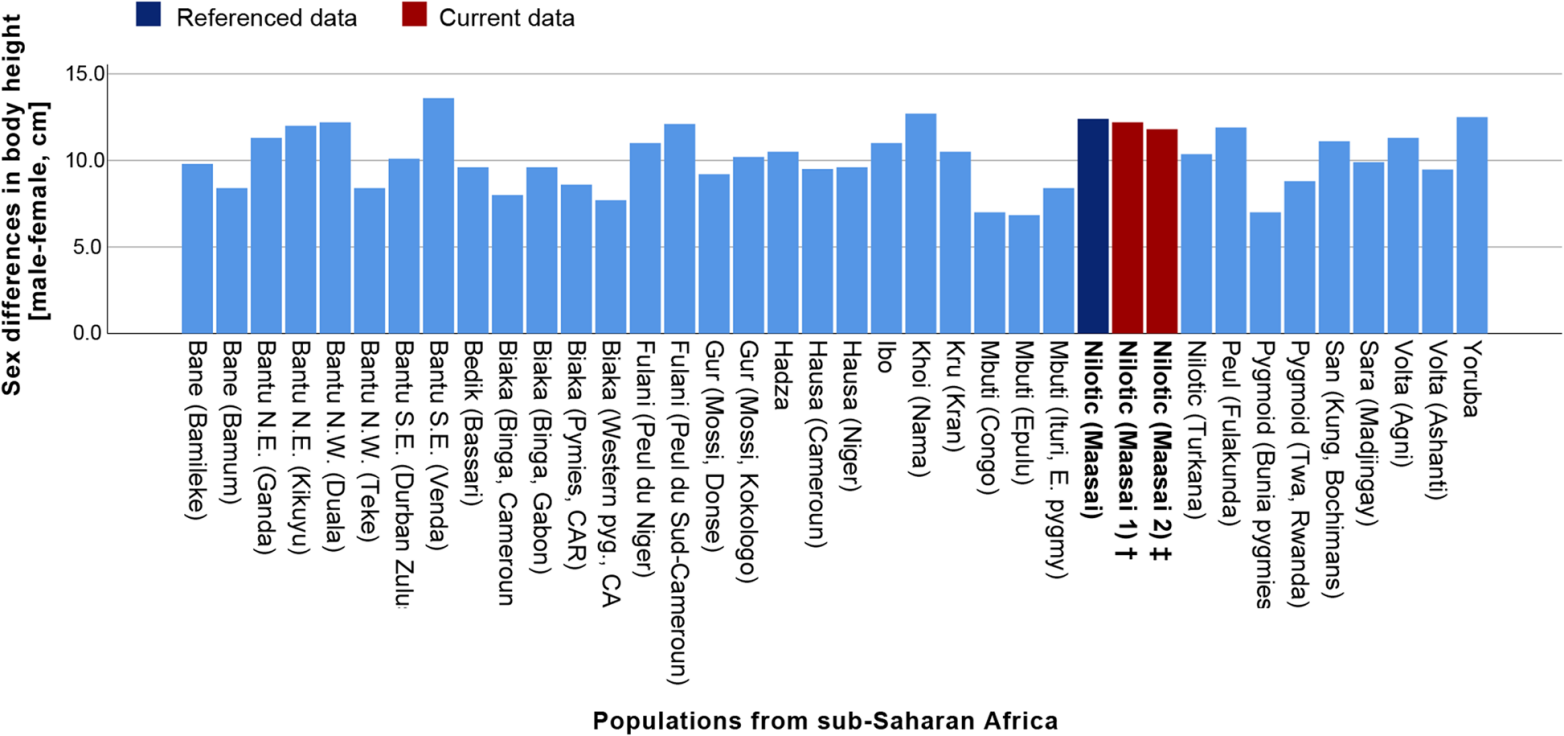
Distribution of sex differences in body height in African sub-Saharan populations, with our data on young- and mid-adults Maasai included. Figure created based on Gustafsson, Lindenfors, 2004 [136]. ^†^ Maasai 1: data from the current sample (age: 17–29 years). ^‡^ Maasai 2: data from the current sample (age: 30–50 years)

Counter to the low facial dimorphism in Maasai, our data revealed quite large body sexual dimorphism, with body height as the most noticeable contributor to it. This parameter together with the three others (under-chest, hips circumferences, and handgrip strength) predicted the sex in Maasai population with 88% of reliability. The comparison of our data on the degree of sexual dimorphism in body height with data on other sub-Saharan populations available, including another Maasai sample [142], confirms that Maasai are among the populations with high level of sex differences in body height in this region (Fig. 10).

Interestingly, effects of static allometry on facial traits of Maasai were mostly defined by the processes of the face growth, rather than related to the growth of the whole body (body height). Earlier studies by other authors focusing on the representatives of European populations come to somewhat contradictive conclusions about the effects of body and face sizes on the facial traits. The study by Mitteroecker with coauthors [46] revealed no significant associations between facial centroid size and facial shape in men, whereas association with body height was significant and explained around 7% of variation in facial traits. Other study in Germans revealed a relation between body height and facial shape, with taller men having narrower and more vertically prolonged lower face [45]. At the same time, the study conducted among Czech students revealed an association between facial size and sexually dimorphic facial traits [143]. The direction of changes in the facial shape with increase of the facial size in Czech study was in line with our findings: larger faces were more prolonged in vertical direction. It was previously demonstrated that head size (head circumference) is correlated with body height in humans [144]. However, correlation between head size and facial shape is expected to be mediated by the factor of the head shape itself. For instance, patterns of integration between facial and basal cranium with respect to brachycephalic and dolichocephalic features may lead to different width-to-height ratios of the faces, which is deeply rooted in human phylogeny [145]. Hence, in the case of dolichocephalic condition, increase of head (and facial) size may result in decrease of fWHRs, which we observed in Maasai. One of the current studies comparing several world populations on facial sexual dimorphism and allometry (measured through body height) suggests that effects of body height on sexual dimorphism of facial shape vary dramatically between people of different descent [38]. In that study, it was demonstrated that both allometric and non-allometric facial sexual dimorphism may largely differ even among populations of common geographical origin (within Africans, Europeans, South-Americans), with African populations generally having very low level of facial sexual dimorphism and its association with body height. The reasons behind such differences remain unclear and deserve to be carefully inspected in the future.

Our results revealed significantly higher wrist diameter in men, and BMI, hips circumference, upper arm circumference, and triceps skinfold in women. Although, according to BMI both men and women Maasai were quite skinny, but women generally had more fat, as judged from triceps skinfolds. Other studies suggest that African men generally have higher percentage of fat-free mass (mainly muscle mass) than women, indicating that under-and-normal weight men likely had a substantial amount of muscle [41, 146], and Maasai men may well fit these assumptions.

Earlier, we have shown the association between physical strength (as measured by HGS) and facial shape for the same Maasai sample [121]. Current study adds more details on these relationships by specifying associations between facial parameters and HGS-related body traits. The strong intrasexual competition in Maasai may be of different intensity in men and women, as well as vary in different age cohorts. Intrasexual competition in men may be most overt during murran stage, as at this time young men establish their social relationships, social status, and gain prestige among peers, while at later stages physical strength may be of less importance compared to social skills (including ability for conflict resolutions and mediation conflicts of others), and successful cattle breeding. Physical strength, which may be a proxy to good health, may be highly important for women during the whole life span, given high energetic costs of pregnancy and breastfeeding, strong competition with other co-wives for resources, and various hard household duties.

Data on healthy and physically active men from western population demonstrate significant relationship between male stature and the change in testosterone levels, and no link between body height and circulating testosterone levels in general. Hence, height may indicate males’ adaptive capabilities to physiologically mobilize their bodies under challenging situations [147]. This may be particularly important in traditional populations, which were not long ago exposed, or still being exposed to raids, carnivorous pressure, and intrasexual contests. Maasai being one of such examples. Some findings suggest that prenatal testosterone exposure may affect the sexually dimorphic facial morphology [17], and androgens in males could contribute to facial sexual dimorphism both before and after puberty [34]. The data from two traditional East African populations, one of which is seminomadic pastoralists similar to Maasai in their ecology and cultural patterns (the Datoga), pointed to the potential role of androgen receptor gene polymorphism in male aggression and reproduction (number of offspring born) [148, 149]. To what extent sexual dimorphism may be a result of selection for attractive partners in Maasai remained a question. In reality, up-till now most marriages in Maasai (especially for women) are arranged by their families. Traditionally, girls being married around the age of 12–13 years, that is during early adolescence, and age differences between spouses are rarely less than 10–15 years. The Maasai remained polygynous, with naturally high fertility profile (M = 5.5 offspring with variation from 2 to 10 per women of post-reproductive age in our sample). High level of pathogen pressure, as well as various environmental threats (including wild animals), remained to be actual selective forces nowadays, and were even more efficient in the past history of Maasai.

Our results demonstrate that facial and body sexual dimorphism may not be directly interrelated in humans, and suggest that in traditional populations, with high level of intrasexual competition among males and high level polygyny body sexual dimorphism, as well as section for height, strength and muscularity may be the men’s priority, whereas facial dimorphism may not be beneficial for survival. It is notable that in populations with large sex differences in body parameters, facial shape differences between men and women can be very low. In recent meta-analysis, Lidborg with co-authors [137] came to the conclusion that body muscularity/strength can be considered sexually selected in human males, and may predict both mating and reproductive success, whereas facial masculinity does not.

We hypothesize that observed body sexual dimorphism in Maasai population may be a compromise between intrasexual competition in males and mate choice selection. Maasai of Ngorongoro remained a population with traditional life-style and natural reproductive profile, muscularity and strength in men continue to be of primary importance for successful cattle breeding, and family protection. While higher body adiposity, as well as waist to hips ratio in women remained to be important prerequisite for successful childbearing and breastfeeding under conditions of limited access to medical help. In fact, earlier we have demonstrated that physically stronger Maasai men were rated as more attractive by women from this same population (see [121]).

It may be highly important to differentiate between body and facial dimorphism and to find the reasons for its’ disproportional expression. The question is what were the driving forces for shaping sexually dimorphic faces and bodies? Selection of female partners with more feminine faces, or selection of younger females? This is especially relevant in the case of traditional populations, where calendar age is not known. Younger looking females are usually more feminine in appearance. This is true both for facial shape as well as body shape [150]. Of sure fertility issues are much more relevant to women than to men, given a limited period till menopause. For men, it is more beneficial to obtain a wife with longer, and potentially more successful reproductive history. In relation to facial ques, as recently demonstrated by Karel Kleisner with co-authors, “ the association between sexual shape dimorphism and attractiveness is moderate for women and weak (or absent) for men. Analysis that distinguishes between allometric and non-allometric components reveals that non-allometric facial dimorphism is preferred in women’s faces but not in faces of men. This might be due to different regimes of ongoing sexual selection acting on men, such as stronger intersexual selection for body height and more intense intrasexual physical competition, compared with women” [38]. Intrasexual competition in men may take direct and indirect forms, but in both cases higher masculinity may be selected for. For instance, in hunter-gatherers, men’s lower (more masculine) voice pitch may be associated with better hunting reputation [151]. But, lower voice pitch may be a proxy to androgen level, while, in turn, facial masculinity and androgen level are positively associated. Another environmental pressure is a high pathogen level, associated with high mortality rate of children. And this factor may be one of the explanations of variations in preference for masculinity observed cross-culturally [152]. Some studies demonstrated that females prefer more masculine male partners, and males, in turn, more feminine looking females in such environment or in the absence of high-quality medical help [153]. It remains to be found out in the future, which sexually dimorphic traits may enhance individual survival probability to different degrees in males and females and show lesser variations between populations of modern humankind [34].

Sociocultural environment may cause substantial pressure on the perception of attractiveness as well. Particularly, facial averageness hypothesis fitted the recently obtained results [154]. Importantly, according to Pavlovič with co-authors, the impact of sexual shape dimorphism on attractiveness was marginal, and found only in Czech European male raters. Earlier, the group of authors presented the data on preferences for sexually dimorphic faces from 12 populations with very diverse levels of economic development [155], and challenge the hypothesis, according to which the “facial dimorphism was an important ancestral signal of heritable mate value.” According to these authors, preferences for facial sexual dimorphism are more evident in large-scale, urban societies, and may be evolutionary novel behavior originated since the time, when people started to interact with large numbers of unfamiliar faces on daily basis.

## Conclusions

Facial shape sex dimorphism in Maasai is very low: sex explained only 1.8% of the total variance. Facial width-to-height ratio (fWHR), measured in six known variants, revealed no significant sex differences. On the contrary, the body sexual dimorphism in Maasai is high, with men being significantly taller, with larger wrist diameter and hand grip strength, and women having higher BMI, hips circumferences, upper arm circumferences, triceps skinfolds. There were practically no associations between facial and body traits, hence, facial and body sexual dimorphisms were not interconnected. The allometric effects on facial traits were mostly related to the face growth, rather than the growth of the whole body. Obtained results clearly demonstrate that under certain conditions the degrees of facial and body sexual dimorphisms may not be interrelated, suggesting different selective processes operating on facial and body sex-specific morphology. Preferences for sexually dimorphic facial and body traits need to be tested cross-culturally in more detail in the future as well.

## Supplementary Information

**Additional file 1: Supplementary Table 1**. Association between facial traits and body height in Maasai (controlled for BMI).

**Additional file 2: Supplementary Table 2**. Association between facial traits and body height in Maasai (without control for BMI).

**Additional file 3: Supplementary Table 3**. Association between facial traits and body parameters in Maasai (controlled for BMI).

## Data Availability

The data that support the findings of this study are available from the corresponding author upon reasonable request.

## Abbreviations

ANCOVA: Analysis of covariance
BMI: Body mass index
COSTECH: Tanzania Commission for Science and Technology
CS: Centroid size
FH: Frankfort Horizontal Plane
fWHR: Facial width-to-height ratio
HGS: Handgrip strength
ICC: Intraclass correlation coefficients
MANCOVA: Multivariate analysis of covariance
NCA: Ngorongoro Conservation Area
SD: Standard deviation
