# Supplementary material for: Facial and body sexual dimorphism are not interconnected in the Maasai
Source: J Physiol Anthropol. 2022 Jan 7;41:3. doi: 10.1186/s40101-021-00276-8 (PMC8740871; doi:10.1186/s40101-021-00276-8)
Supplement: Supplementary file 2 — Additional file 2: Supplementary Table 2. Association between facial traits and body height in Maasai (without control for BMI). [file 40101_2021_276_MOESM2_ESM.docx]

**Supplementary Table 2.** Association between facial traits and body height in Maasai (without control for BMI)

| **Predictor** | **Dependent variables** | **Definition** | **Partial Eta^2^** | **Sig.** |
| --- | --- | --- | --- | --- |
| Men | | | | |
| Body height | Upper fWHR | \|Zy-Zy\| / \|N-Sto\| | 0.001 | 0.781 |
|  |  | \|Zy-Zy\| / \|Gl-Sto\| | 0.005 | 0.406 |
|  |  | \|Zy-Zy\| / \|N-Ls\| | <0.001 | 0.875 |
|  |  | \|Zy-Zy\| / \|Gl-Ls\| | 0.010 | 0.255 |
|  | Total fWHR | \|Zy-Zy\| / \|N-Gn\| | 0.024 | 0.073 |
|  | Lower fWHR | \|Zy-Zy\| / \|Sn-Gn\| | 0.022 | 0.081 |
|  | Cheekbone prominence | \|Zy-Zy\| / \|Go-Go\| | 0.008 | 0.315 |
|  | Mandibular index | \|Go-Go\| / \|Sto-Gn\| | 0.028 | 0.050 |
|  | Nasal index | \|Al-Al\| / \|N-Sn\| | <0.001 | 0.963 |
|  | Mouth shape | \|Ls-Li\| / \|Ch-Ch\| | <0.001 | 0.843 |
|  | Mouth-face index | \|Ch-Ch\| / \|Zy-Zy\| | 0.010 | 0.238 |
|  | Height-to-width ratio of the eye (mean) | \|Ps-Pi\| / \|Ex-En\| | <0.001 | 0.861 |
| Women |  |  |  |  |
| Body height | Upper fWHR | \|Zy-Zy\| / \|N-Sto\| | 0.024 | 0.127 |
|  |  | \|Zy-Zy\| / \|Gl-Sto\| | 0.051 | 0.027* |
|  |  | \|Zy-Zy\| / \|N-Ls\| | 0.010 | 0.338 |
|  |  | \|Zy-Zy\| / \|Gl-Ls\| | 0.035 | 0.068 |
|  | Total fWHR | \|Zy-Zy\| / \|N-Gn\| | 0.058 | 0.018* |
|  | Lower fWHR | \|Zy-Zy\| / \|Sn-Gn\| | 0.051 | 0.026 |
|  | Cheekbone prominence | \|Zy-Zy\| / \|Go-Go\| | <0.001 | 0.919 |
|  | Mandibular index | \|Go-Go\| / \|Sto-Gn\| | 0.027 | 0.105 |
|  | Nasal index | \|Al-Al\| / \|N-Sn\| | 0.017 | 0.208 |
|  | Mouth shape | \|Ls-Li\| / \|Ch-Ch\| | 0.013 | 0.261 |
|  | Mouth-face index | \|Ch-Ch\| / \|Zy-Zy\| | <0.001 | 0.845 |
|  | Height-to-width ratio of the eye (mean) | \|Ps-Pi\| / \|Ex-En\| | 0.001 | 0.726 |

MANCOVA results are presented. Dependent variables: facial traits; independent variable: body height. Definitions of the facial landmarks used for facial traits calculation can be found in the main text (Fig. 1). Weakly significant association, which does not survive Bonferroni correction for multiple testing are marked with *.
