# Supplementary material for: Facial and body sexual dimorphism are not interconnected in the Maasai
Source: J Physiol Anthropol. 2022 Jan 7;41:3. doi: 10.1186/s40101-021-00276-8 (PMC8740871; doi:10.1186/s40101-021-00276-8)
Supplement: Supplementary file 3 — Additional file 3: Supplementary Table 3. Association between facial traits and body parameters in Maasai (controlled for BMI). [file 40101_2021_276_MOESM3_ESM.docx]

**Supplementary Table 3.** Association between facial traits and body parameters in Maasai (controlled for BMI)

| **Predictor** | **Dependent variables** | **Definition** | **Partial Eta^2^** | **Sig.** |
| --- | --- | --- | --- | --- |
| Men | | | | |
| Wrist diameter | Upper fWHR | \|Zy-Zy\| / \|N-Sto\| | 0.004 | 0.497 |
|  |  | \|Zy-Zy\| / \|Gl-Sto\| | 0.001 | 0.671 |
|  |  | \|Zy-Zy\| / \|N-Ls\| | 0.001 | 0.743 |
|  |  | \|Zy-Zy\| / \|Gl-Ls\| | 0.011 | 0.252 |
|  | Total fWHR | \|Zy-Zy\| / \|N-Gn\| | 0.015 | 0.181 |
|  | Lower fWHR | \|Zy-Zy\| / \|Sn-Gn\| | 0.014 | 0.190 |
|  | Cheekbone prominence | \|Zy-Zy\| / \|Go-Go\| | 0.009 | 0.295 |
|  | Mandibular index | \|Go-Go\| / \|Sto-Gn\| | 0.005 | 0.453 |
|  | Nasal index | \|Al-Al\| / \|N-Sn\| | 0.003 | 0.556 |
|  | Mouth shape | \|Ls-Li\| / \|Ch-Ch\| | 0.015 | 0.179 |
|  | Mouth-face index | \|Ch-Ch\| / \|Zy-Zy\| | <0.001 | 0.852 |
|  | Height-to-width ratio of the eye (mean) | \|Ps-Pi\| / \|Ex-En\| | 0.004 | 0.469 |
| Upper arm circumference | Upper fWHR | \|Zy-Zy\| / \|N-Sto\| | 0.075 | **0.002*** |
|  |  | \|Zy-Zy\| / \|Gl-Sto\| | 0.050 | 0.011 |
|  |  | \|Zy-Zy\| / \|N-Ls\| | 0.046 | 0.016 |
|  |  | \|Zy-Zy\| / \|Gl-Ls\| | 0.033 | 0.041 |
|  | Total fWHR | \|Zy-Zy\| / \|N-Gn\| | 0.009 | 0.283 |
|  | Lower fWHR | \|Zy-Zy\| / \|Sn-Gn\| | 0.005 | 0.443 |
|  | Cheekbone prominence | \|Zy-Zy\| / \|Go-Go\| | 0.067 | **0.003*** |
|  | Mandibular index | \|Go-Go\| / \|Sto-Gn\| | 0.003 | 0.535 |
|  | Nasal index | \|Al-Al\| / \|N-Sn\| | 0.033 | 0.041 |
|  | Mouth shape | \|Ls-Li\| / \|Ch-Ch\| | 0.022 | 0.095 |
|  | Mouth-face index | \|Ch-Ch\| / \|Zy-Zy\| | 0.016 | 0.157 |
|  | Height-to-width ratio of the eye (mean) | \|Ps-Pi\| / \|Ex-En\| | 0.002 | 0.618 |
| Under-chest circumference | Upper fWHR | \|Zy-Zy\| / \|N-Sto\| | 0.015 | 0.171 |
|  |  | \|Zy-Zy\| / \|Gl-Sto\| | 0.022 | 0.096 |
|  |  | \|Zy-Zy\| / \|N-Ls\| | 0.004 | 0.500 |
|  |  | \|Zy-Zy\| / \|Gl-Ls\| | 0.009 | 0.277 |
|  | Total fWHR | \|Zy-Zy\| / \|N-Gn\| | 0.010 | 0.260 |
|  | Lower fWHR | \|Zy-Zy\| / \|Sn-Gn\| | 0.001 | 0.774 |
|  | Cheekbone prominence | \|Zy-Zy\| / \|Go-Go\| | 0.073 | **0.002*** |
|  | Mandibular index | \|Go-Go\| / \|Sto-Gn\| | 0.020 | 0.114 |
|  | Nasal index | \|Al-Al\| / \|N-Sn\| | 0.007 | 0.350 |
|  | Mouth shape | \|Ls-Li\| / \|Ch-Ch\| | 0.030 | 0.049 |
|  | Mouth-face index | \|Ch-Ch\| / \|Zy-Zy\| | 0.022 | 0.093 |
|  | Height-to-width ratio of the eye (mean) | \|Ps-Pi\| / \|Ex-En\| | 0.002 | 0.612 |
| Hip circumference | Upper fWHR | \|Zy-Zy\| / \|N-Sto\| | 0.008 | 0.308 |
|  |  | \|Zy-Zy\| / \|Gl-Sto\| | 0.013 | 0.199 |
|  |  | \|Zy-Zy\| / \|N-Ls\| | <0.001 | 0.965 |
|  |  | \|Zy-Zy\| / \|Gl-Ls\| | 0.001 | 0.728 |
|  | Total fWHR | \|Zy-Zy\| / \|N-Gn\| | 0.001 | 0.710 |
|  | Lower fWHR | \|Zy-Zy\| / \|Sn-Gn\| | 0.002 | 0.641 |
|  | Cheekbone prominence | \|Zy-Zy\| / \|Go-Go\| | 0.013 | 0.200 |
|  | Mandibular index | \|Go-Go\| / \|Sto-Gn\| | 0.009 | 0.299 |
|  | Nasal index | \|Al-Al\| / \|N-Sn\| | 0.003 | 0.512 |
|  | Mouth shape | \|Ls-Li\| / \|Ch-Ch\| | 0.046 | 0.015 |
|  | Mouth-face index | \|Ch-Ch\| / \|Zy-Zy\| | 0.003 | 0.509 |
|  | Height-to-width ratio of the eye (mean) | \|Ps-Pi\| / \|Ex-En\| | 0.009 | 0.279 |
| Women | | | | |
| Wrist diameter | Upper fWHR | \|Zy-Zy\| / \|N-Sto\| | 0.032 | 0.104 |
|  |  | \|Zy-Zy\| / \|Gl-Sto\| | 0.001 | 0.819 |
|  |  | \|Zy-Zy\| / \|N-Ls\| | 0.022 | 0.182 |
|  |  | \|Zy-Zy\| / \|Gl-Ls\| | <0.001 | 0.904 |
|  | Total fWHR | \|Zy-Zy\| / \|N-Gn\| | 0.006 | 0.467 |
|  | Lower fWHR | \|Zy-Zy\| / \|Sn-Gn\| | 0.001 | 0.758 |
|  | Cheekbone prominence | \|Zy-Zy\| / \|Go-Go\| | 0.002 | 0.667 |
|  | Mandibular index | \|Go-Go\| / \|Sto-Gn\| | 0.010 | 0.368 |
|  | Nasal index | \|Al-Al\| / \|N-Sn\| | 0.001 | 0.832 |
|  | Mouth shape | \|Ls-Li\| / \|Ch-Ch\| | 0.001 | 0.828 |
|  | Mouth-face index | \|Ch-Ch\| / \|Zy-Zy\| | 0.044 | 0.056 |
|  | Height-to-width ratio of the eye (mean) | \|Ps-Pi\| / \|Ex-En\| | 0.009 | 0.392 |
| Upper arm circumference | Upper fWHR | \|Zy-Zy\| / \|N-Sto\| | 0.029 | 0.109 |
|  |  | \|Zy-Zy\| / \|Gl-Sto\| | 0.009 | 0.363 |
|  |  | \|Zy-Zy\| / \|N-Ls\| | 0.019 | 0.196 |
|  |  | \|Zy-Zy\| / \|Gl-Ls\| | 0.005 | 0.489 |
|  | Total fWHR | \|Zy-Zy\| / \|N-Gn\| | 0.012 | 0.299 |
|  | Lower fWHR | \|Zy-Zy\| / \|Sn-Gn\| | 0.003 | 0.609 |
|  | Cheekbone prominence | \|Zy-Zy\| / \|Go-Go\| | 0.062 | 0.018 |
|  | Mandibular index | \|Go-Go\| / \|Sto-Gn\| | 0.008 | 0.396 |
|  | Nasal index | \|Al-Al\| / \|N-Sn\| | 0.119 | **0.001*** |
|  | Mouth shape | \|Ls-Li\| / \|Ch-Ch\| | <0.001 | 0.927 |
|  | Mouth-face index | \|Ch-Ch\| / \|Zy-Zy\| | 0.011 | 0.320 |
|  | Height-to-width ratio of the eye (mean) | \|Ps-Pi\| / \|Ex-En\| | 0.003 | 0.615 |
| Triceps skinfold | Upper fWHR | \|Zy-Zy\| / \|N-Sto\| | 0.030 | 0.102 |
|  |  | \|Zy-Zy\| / \|Gl-Sto\| | 0.020 | 0.187 |
|  |  | \|Zy-Zy\| / \|N-Ls\| | 0.018 | 0.213 |
|  |  | \|Zy-Zy\| / \|Gl-Ls\| | 0.012 | 0.308 |
|  | Total fWHR | \|Zy-Zy\| / \|N-Gn\| | 0.043 | 0.050 |
|  | Lower fWHR | \|Zy-Zy\| / \|Sn-Gn\| | 0.025 | 0.140 |
|  | Cheekbone prominence | \|Zy-Zy\| / \|Go-Go\| | 0.001 | 0.830 |
|  | Mandibular index | \|Go-Go\| / \|Sto-Gn\| | 0.020 | 0.181 |
|  | Nasal index | \|Al-Al\| / \|N-Sn\| | 0.021 | 0.178 |
|  | Mouth shape | \|Ls-Li\| / \|Ch-Ch\| | 0.001 | 0.782 |
|  | Mouth-face index | \|Ch-Ch\| / \|Zy-Zy\| | 0.028 | 0.114 |
|  | Height-to-width ratio of the eye (mean) | \|Ps-Pi\| / \|Ex-En\| | 0.008 | 0.414 |
| Under-chest circumference | Upper fWHR | \|Zy-Zy\| / \|N-Sto\| | 0.001 | 0.719 |
|  |  | \|Zy-Zy\| / \|Gl-Sto\| | 0.001 | 0.791 |
|  |  | \|Zy-Zy\| / \|N-Ls\| | 0.001 | 0.724 |
|  |  | \|Zy-Zy\| / \|Gl-Ls\| | 0.001 | 0.828 |
|  | Total fWHR | \|Zy-Zy\| / \|N-Gn\| | 0.017 | 0.216 |
|  | Lower fWHR | \|Zy-Zy\| / \|Sn-Gn\| | 0.004 | 0.537 |
|  | Cheekbone prominence | \|Zy-Zy\| / \|Go-Go\| | 0.006 | 0.487 |
|  | Mandibular index | \|Go-Go\| / \|Sto-Gn\| | 0.071 | 0.011 |
|  | Nasal index | \|Al-Al\| / \|N-Sn\| | 0.033 | 0.086 |
|  | Mouth shape | \|Ls-Li\| / \|Ch-Ch\| | 0.007 | 0.449 |
|  | Mouth-face index | \|Ch-Ch\| / \|Zy-Zy\| | 0.002 | 0.658 |
|  | Height-to-width ratio of the eye (mean) | \|Ps-Pi\| / \|Ex-En\| | 0.048 | 0.038 |
| Hip circumference | Upper fWHR | \|Zy-Zy\| / \|N-Sto\| | 0.010 | 0.352 |
|  | Total fWHR | \|Zy-Zy\| / \|Gl-Sto\| | 0.071 | 0.013 |
|  |  | \|Zy-Zy\| / \|N-Ls\| | 0.001 | 0.728 |
|  |  | \|Zy-Zy\| / \|Gl-Ls\| | 0.049 | 0.043 |
|  |  | \|Zy-Zy\| / \|N-Gn\| | 0.056 | 0.030 |
|  | Lower fWHR | \|Zy-Zy\| / \|Sn-Gn\| | 0.048 | 0.044 |
|  | Cheekbone prominence | \|Zy-Zy\| / \|Go-Go\| | <0.001 | 0.875 |
|  | Mandibular index | \|Go-Go\| / \|Sto-Gn\| | 0.041 | 0.064 |
|  | Nasal index | \|Al-Al\| / \|N-Sn\| | 0.011 | 0.344 |
|  | Mouth shape | \|Ls-Li\| / \|Ch-Ch\| | 0.020 | 0.199 |
|  | Mouth-face index | \|Ch-Ch\| / \|Zy-Zy\| | 0.004 | 0.585 |
|  | Height-to-width ratio of the eye (mean) | \|Ps-Pi\| / \|Ex-En\| | 0.001 | 0.762 |

MANCOVA results are presented. Dependent variables: facial traits; independent variables: 1) BMI, 2) each of body parameters. Effect size (partial Eta^2^) and significance level (p) are presented only for each body parameter (after controlling for BMI). Definitions of the facial landmarks used for facial traits calculation can be found in Fig. 1. Significant associations, which survived Bonferroni correction for 12 tests are marked with *, and presented in bold.
